# Non-surgical management in children with non-refluxing primary megaureter: a systematic review and meta-analysis

**DOI:** 10.1007/s00467-023-05938-6

**Published:** 2023-03-30

**Authors:** Kathrin Buder, Kathrin Opherk, Sara Mazzi, Katharina Rohner, Marcus Weitz

**Affiliations:** 1 Department of General Pediatrics and Hematology/Oncology, University Hospital Tübingen, University Children’s Hospital, Hoppe-Seyler-Str. 1, D – 72076 Tübingen, Germany; 2 Pediatric Nephrology Department, University Children’s Hospital Zurich, Steinwiesstr. 75, CH – 8032 Zurich, Switzerland

**Keywords:** Primary megaureter, Uretero-vesical junction obstruction, Pelvicalyceal dilatation, Differential renal function, Non-surgical management

## Abstract

**Background:**

Children with non-refluxing primary megaureter are mostly managed by a watchful approach with close follow-up and serial imaging.

**Objectives:**

This systematic review and meta-analysis aimed to determine whether there is sufficient evidence to support the current non-surgical management strategy in these patients.

**Data sources:**

A comprehensive search including electronic literature databases, clinical trial registries, and conference proceedings was performed.

**Data synthesis methods:**

Outcomes were estimated as pooled prevalence. If meta-analytical calculations were not appropriate, outcomes were provided in a descriptive manner.

**Results:**

Data from 8 studies (290 patients/354 renal units) were included. For the primary outcome, differential renal function estimated by functional imaging, meta-analysis was impossible due to reported data not being precise. Pooled prevalence for secondary surgery was 13% (95% confidence interval: 8–19%) and for resolution 61% (95% confidence interval: 42–78%). The risk of bias was moderate or high in most studies.

**Limitations:**

This analysis was limited by the low number of eligible studies with few participants and high clinical heterogeneity, and the poor quality of the available data.

**Conclusions:**

The low pooled prevalence of secondary surgical intervention and high pooled prevalence of resolution may support the current non-surgical management in children with non-refluxing primary megaureter. However, these results should be interpreted cautiously due to the limited available body of evidence. Future studies should overcome existing limitations of imaging methods by using standardized, comparable criteria and report outcome parameters in a quantitative manner. This would allow more sufficient data synthesis to provide evidence-based recommendations for clinical decision-making and counseling.

**Systematic review registration:**

The protocol was registered on PROSPERO under CRD42019134502.

**Supplementary information:**

The online version contains supplementary material available at 10.1007/s00467-023-05938-6.

## Introduction

Among congenital uropathies, primary megaureter (PM) represents a frequent condition [1–3]. The descriptive term refers to an enlarged ureteral diameter with or without associated pelvicalyceal dilatation due to an anomaly at the vesicoureteral junction [1, 2]. Primary megaureters are categorized into refluxing, non-refluxing, obstructive, and non-obstructive which affects clinical management [4, 5]. The management of refluxing PM is mostly affected by the grade and clinical presentation of vesicoureteral reflux [6]. In non-refluxing PM, deteriorated differential renal function (DRF) and/or symptoms attributed to potential impaired urinary drainage are decisive for the management strategy [1, 7–9]. Spontaneous regression rates up to 70–80% have been observed in non-refluxing PM, leading to a paradigm change toward a primary non-surgical approach within the last three decades [1, 3, 10, 11]. However, clinical decision-making and management in affected children is still challenging. This is due to ongoing diagnostic difficulties to identify significant obstructions which might result in non-restorable kidney function deterioration [1, 3, 10]. Therefore, a timely indication for secondary surgical intervention, i.e. surgical treatment following a primary watchful approach, is often not possible [1, 7]. This systematic review and meta-analysis aimed to evaluate the current evidence on primary non-surgical management in children with non-refluxing PM.

## Material and methods

The protocol for this systematic review was registered in PROSPERO (http://www.crd.york.ac.uk/PROSPERO) under registration number CRD42019134502. The Preferred Reporting Items for Systematic Reviews and Meta-Analyses (PRISMA) statement and items of the Cochrane Handbook for Systematic Reviews of Interventions were used for reporting [12, 13].

## Eligibility criteria

Children and adolescents aged < 18 years meeting the following eligibility criteria were included:

1. Postnatal diagnosis of PM, defined as ureteral dilatation ≥ 7 mm or descriptive information about an enlarged ureter referred to as a megaureter, with or without associated pelvicalyceal dilatation, assessed by ultrasound or any other appropriate cross-sectional imaging method at the age of ≥ 7 days;
2. Exclusion of vesicoureteral reflux by voiding cystourethrography or voiding urosonography;
3. Reported serial DRF and urinary drainage measurements examined by (technetium-99 m-dimercaptosuccinyl-acid (^99m^Tc-DMSA), technetium-99 m-diethylenetriamine-pentaacetic-acid (^99m^Tc-DTPA), and technetium-99 m-mercaptoacetyltriglycine (^99m^Tc-MAG3)) renal scintigraphy or magnetic resonance urography.

Studies including pediatric patients with secondary megaureter(s), ipsilateral concomitant urinary tract or kidney anomalies other than PM (e.g., duplex kidney, horseshoe kidney), ipsilateral comorbidities affecting urinary drainage (e.g., uronephrolithiasis), primary surgical interventions without previous surveillance resulting from imaging findings, balloon dilatation, and ureteral stenting, as well as case reports and series with ≤ 3 participants were excluded. In case of mixed study populations, only the data of patients fulfilling the inclusion criteria were extracted. Studies including patients with bilateral non-refluxing PM were not excluded if data for selected outcomes were extractable.

## Outcomes

The primary outcome was the change of DRF in children with non-refluxing PM and a primary non-surgical management. Secondary outcomes included (1) urinary drainage; (2) deterioration, persistence, improvement, or resolution of ureteral and/or pelvicalyceal dilatation; (3) secondary surgical intervention (defined as surgical treatment during follow-up after a primary watchful approach); (4) symptoms associated with non-refluxing PM; (5) adverse effects (e.g., frequency of analgo-sedation, radiation exposure); (6) costs of intervention; and (7) health-related quality of life.

Considering the lack of uniform reporting in terms of definitions, classification systems, and interpretation of functional imaging findings, data were extracted as reported by the studies.

## Data sources and searches

A highly sensitive search strategy was developed to obtain all studies reporting on PM in children (Appendix 1). The literature search was carried out without language restriction in the following electronic databases: EMBASE/Embase (1947 to July 21, 2021), MEDLINE/Ovid (1946 through July 21, 2021), the Cochrane Central Register of Controlled Trials (CENTRAL) and the Central Database of Systematic Reviews (CDSR, Issue 7, 2021), with an update limited to MEDLINE/Ovid and CENTRAL/CDSR on September 27, 2022 (due to missing license for an updated EMBASE/Embase search). In addition, clinical trial registries and conference proceedings were searched to identify ongoing or recently completed trials and links to other related databases and resources (Appendix 2). The reference lists of articles relevant to this review were also inspected for trials and publications.

## Study selection

Abstracts and titles obtained from the searches were screened for eligible studies and full texts of identified studies analyzed by two reviewers independently. Disagreements were resolved by discussion or consensus involving a third author. For studies reporting the results in more than one publication, the most recent and nonredundant data were included. If additional information was required, the corresponding authors of the articles were contacted. Excluded studies are listed with reasons (Appendix 3).

## Data extraction

Data extraction was undertaken independently by two authors using a standard extraction form, and discrepancies were resolved in consultation or by discussion with a third party. In case of studies reporting separate subpopulations (e.g., refluxing and non-refluxing PM), individuals meeting the inclusion criteria were extracted separately.

## Quality assessment

To ensure comparability and integrative analysis, the risk of bias (ROB) was evaluated and summarized for each study by two independent reviewers, using the Cochrane risk of bias tool for non-randomized studies (ROBINS-I; https://sites.google.com/site/riskofbiastool/welcome/home/current-version-of-robins-i/robins-i-template-2016) [14, 15]. For reasons of simplification and standardization, this well-established tool was used as a deviation from the original PROSPERO protocol.

## Data synthesis and analysis

The outcomes were calculated from the number of affected renal units (numerator) and the total number of renal units diagnosed with non-refluxing PM in each study (denominator). Varying denominators resulted from the fact that several data points and outcomes were not provided by some trials. The focus was set on renal units instead of individuals, because all studies enrolled patients with bilateral non-refluxing PM. For the pre-defined outcomes, the pooled prevalence (in terms of proportion) using a random effects model and generalized linear mixed model (i.e. a random intercept logistic regression model with logit transformation) was calculated [16]. The confidence interval (CI) for pooled prevalence was estimated as the normal approximation interval at the 95% level. *I*^2^ values were interpreted as presenting moderate (30–60%), substantial (50–90%), or considerable (75–100%) heterogeneity [13, 17]. Sensitivity analyses were performed to analyze the causes of heterogeneity and the statistical effects of study designs. Funnel plots were carried out to investigate publication bias [18]. All data were entered into R 4.2.1 for statistical analysis, with the additional package meta 6.0–0, and ROB was visualized by using robvis [15, 19]. Imprecise or missing data of pre-defined outcomes not usable for statistical analysis were reported in a descriptive or narrative manner.

## Results

### Search results

**Fig. 1:**
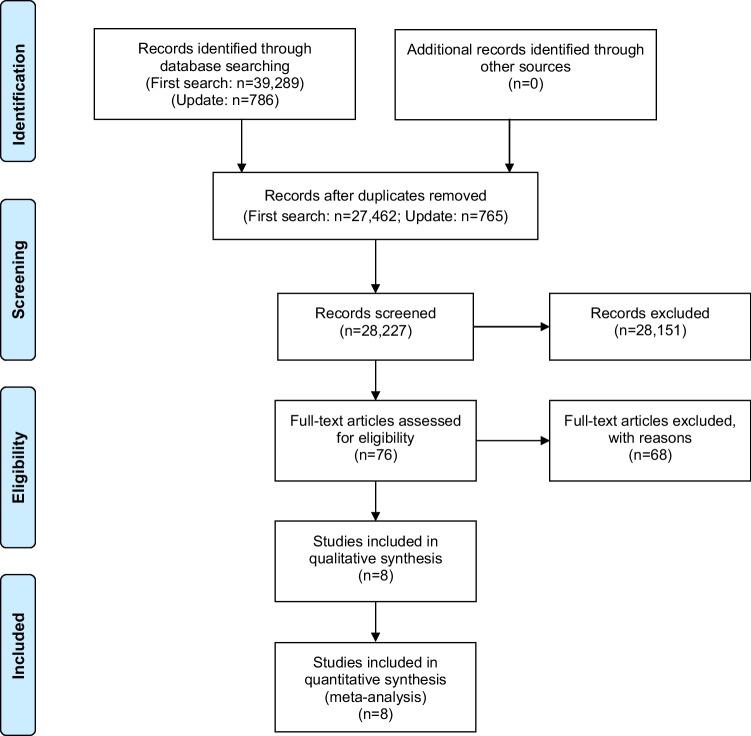
Study flow diagram according to the Preferred Reporting Items for Systematic Reviews and Meta-Analyses (PRISMA) statement [12]

The search yielded 28,227 records, of which 76 were screened by full text (Fig. 1). Of these, 68 were excluded with reasons (Appendix 3), resulting in 8 studies eligible for this review. The search in conference proceedings, clinical trial registries, reference lists of relevant articles, or personal contacts did not identify additional studies.

### Study and patient characteristics

The included 8 studies enrolled 309 patients (77% [239/309] males) with 375 renal units. Of these 375 renal units, 21 (*n* = 19 patients) were excluded due to primary surgical intervention, leaving 354 renal units (*n* = 290 patients) for analysis.

The 8 studies were monocentric, predominantly conducted with a retrospective study design (75% [6/8]) at European tertiary centers (88% [7/8]) between 1981 and 2013 and published beyond 2000 (63% [5/8]). Detailed study characteristics are listed in Appendix 4. Primary megaureter was diagnosed prenatally and confirmed postnatally in 68% (209/309), during the neonatal period in 13% (39/309), and incidentally or symptomatically in 20% (61/309) (range, 3–108 months). Twenty percent (63/309) had bilateral PM. Follow-up time ranged from 5 to 180 months, and covered periods of at least 24 months in 2 [20, 21], and of at least 12 months in 4 studies [22–25].

#### Functional imaging

For assessment of DRF and urinary drainage predominantly ^99m^Tc-DMSA, ^99m^Tc-DTPA, or ^99m^Tc-MAG3 renal scintigraphy was conducted (Appendices 4 and 5). Diagnostic findings at study enrolment are summarized in Appendix 6. Decreased DRF was defined as ≤ 40% in 5 [21, 23, 25–27], < 45% in one [24], and not specified in 2 studies [20, 22]. At baseline, 96% (119/124; 4 studies) of non-refluxing PMs had a DRF ≥ 40% [20, 21, 25, 26]. Urinary drainage was evaluated in terms of urinary drainage pattern in 3 [24, 26, 27], washout time (clearance half time or time to clear up 75%) in 2 studies [21, 23], a combination of both in one study [25], and not specified in 2 [20, 22]. Urinary drainage pattern and/or clearance time indicated obstruction in 11% (17/153; 4 studies) and partial/intermediate/equivocal obstruction in 35% (54/153) of PMs; the remaining renal units (54% [82/153) were graded as dilated non-obstructive/ functionally obstructive or non-obstructive/ normal (Appendix 6) [24–27].

#### Ureteral and pelvicalyceal dilatation

Across all studies, imaging methods to diagnose ureteral and pelvicalyceal dilatation were comparable (e.g., ultrasound, intravenous pyelogram), but definitions, classification systems, and interpretations were inconsistent (Appendix 5).

#### Criteria for primary surgical versus primary non-surgical management

Criteria for initial non-surgical treatment were often not precisely reported. Indications for primary surgical intervention are listed in Appendix 7.

### Quality assessment

**Fig. 2:**
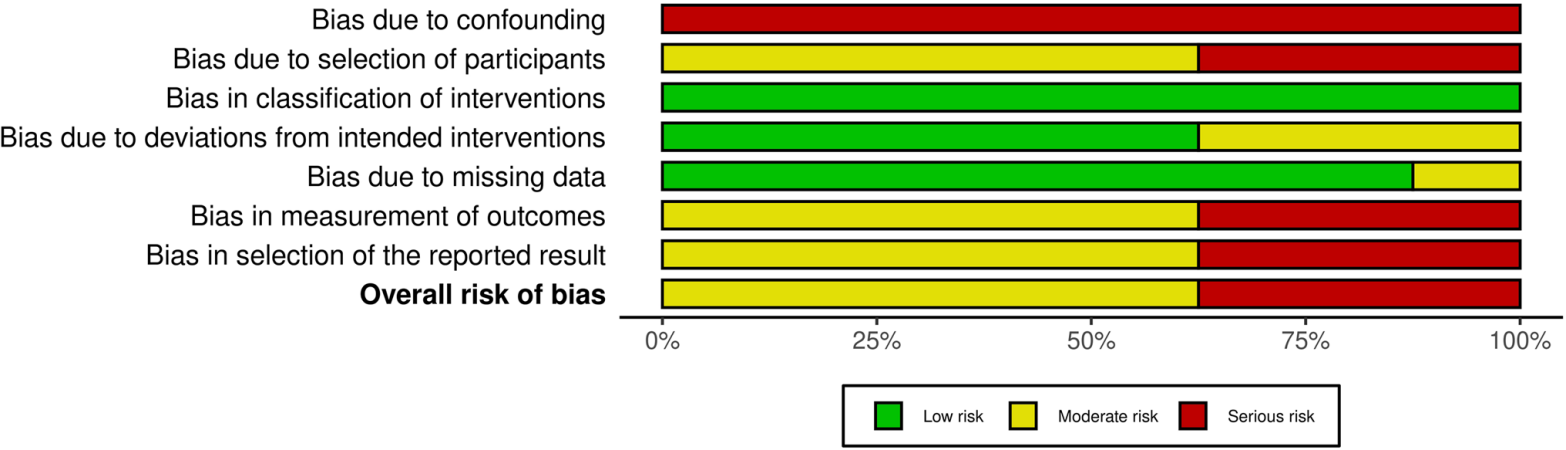
Risk of bias summary: review authors’ judgements on each risk of bias category presented as percentages across all studies included

**Fig. 3:**
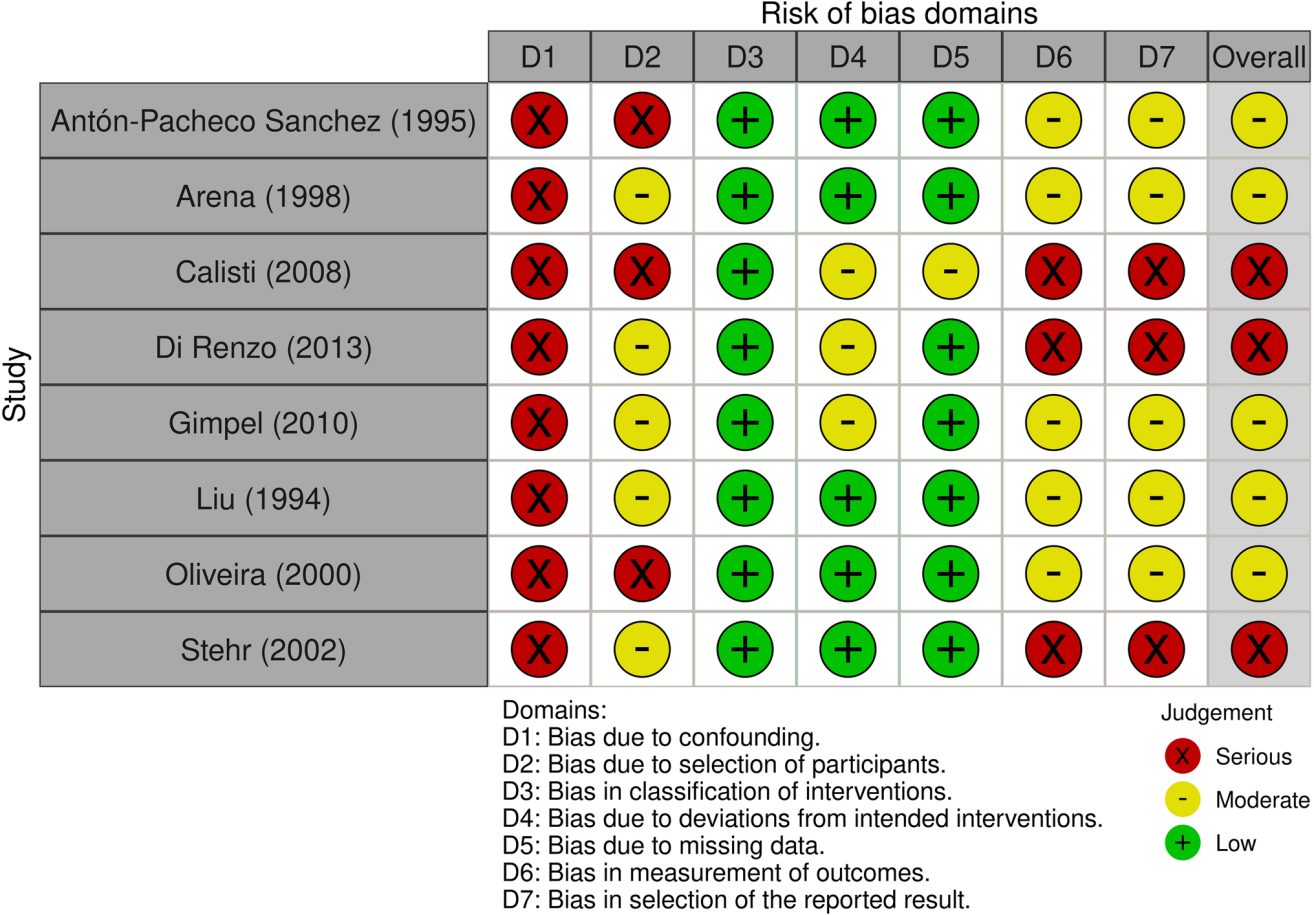
Risk of bias: review authors’ judgements on each risk of bias category for each study included

The ROB was assessed for each study and across all studies and categorized into low, moderate, and high (Figs. 2 and 3). The domain “confounders” was classified as being at serious risk in all studies. The domains “selection of participants,” “measurement of outcomes,” and “selection of reported results,” were considered presenting a moderate or serious risk in the vast majority of the studies. Overall, 3 out of 8 studies had a high ROB.

### Primary outcome

#### Differential renal function during follow-up

Serial DRF assessment was often reported only in a descriptive manner (e.g., “stable”), and partially provided only for selected participants (Appendix 8) impeding meta-analytical calculations. Deterioration of DRF was indicated in 4% (11/252; 6 studies) of non-refluxing PMs. Data on DRF course in patients undergoing secondary surgical intervention were not appropriate for statistical analysis (Appendix 9).

### Secondary outcomes

#### Urinary drainage during follow-up

Urinary drainage at follow-up was not documented in a standardized manner with extractable data allowing meta-analytical calculations (Appendix 8). In 8% (7/89; 3 studies) of non-refluxing PM, urinary drainage deterioration was observed [20, 24, 25] (Appendix 8).

#### Resolution of non-refluxing primary megaureter with/without pelvicalyceal dilatation

**Fig. 4:**
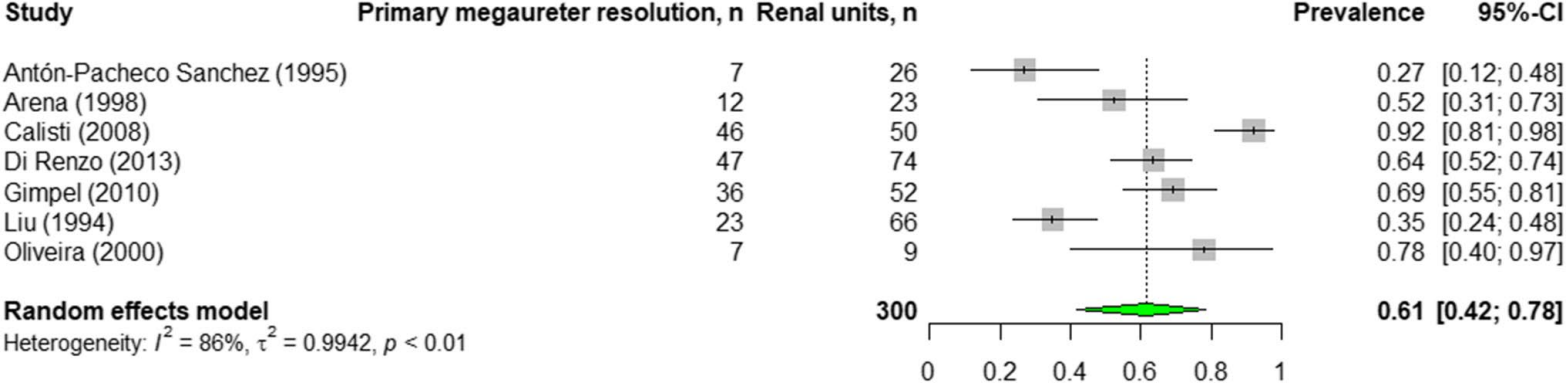
Pooled prevalence of resolution in non-refluxing primary megaureter

The pooled prevalence for resolution of non-refluxing PM with and without pelvicalyceal dilatation as defined by the studies was 61% (95% CI: 42–78%; *n* = 300; range, 27% [25] to 92% [22]; 7 studies) with a considerable heterogeneity (*I*^2^ = 86%, *p* < 0.01) (Fig. 4 and Appendices 5 and 8).

#### Secondary surgical intervention

**Fig. 5:**
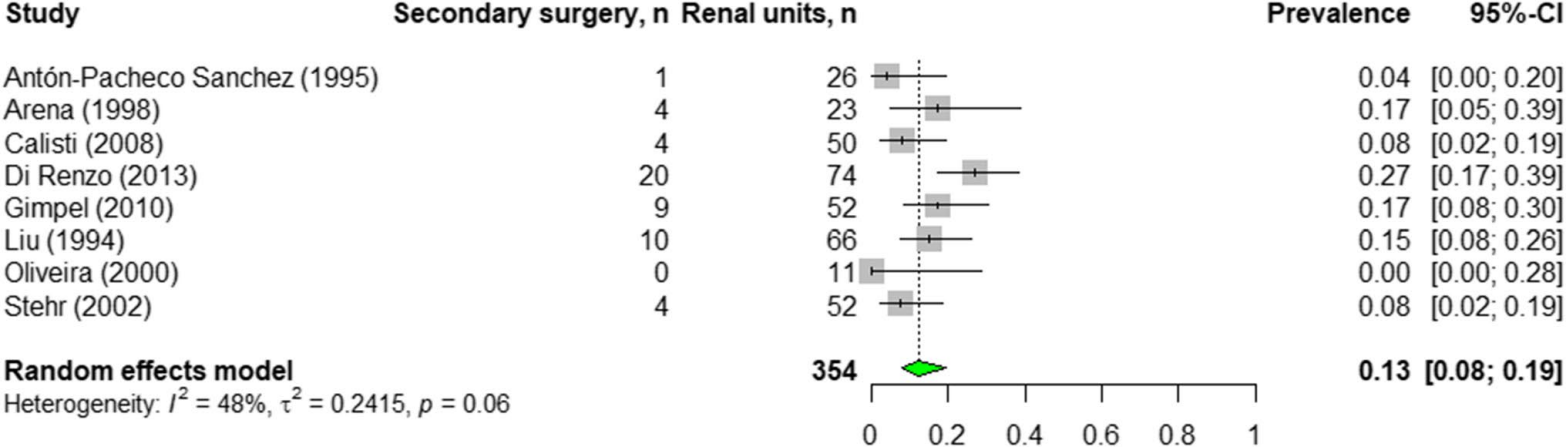
Pooled prevalence of secondary surgery in non-refluxing primary megaureter

The pooled prevalence of secondary surgical intervention was 13% (95% CI: 8–19%; *n* = 354; range, 0% [20] to 27% [21]; 8 studies) with a moderate heterogeneity (*I*^2^ = 48%, *p* = 0.06) (Fig. 5 and Appendix 8).

**Fig. 6:**
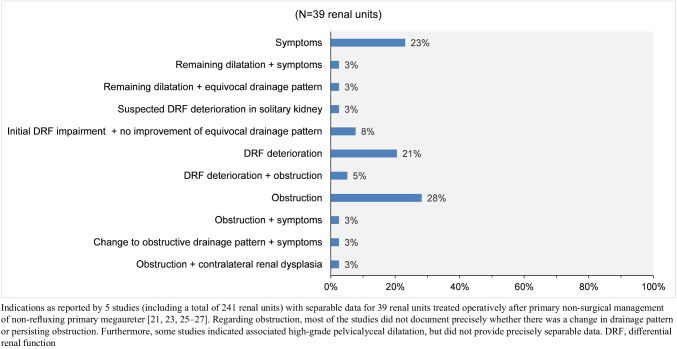
Reasons for secondary surgery in non-refluxing primary megaureter

Selection criteria and indications for secondary surgical intervention are displayed in Fig. 6 and Appendix 7. Secondary surgical intervention was carried out in a range from 6 to 96 months after diagnosis (7 studies) [21–27].

#### Symptoms reported in non-refluxing primary megaureter

Some studies reported clinical symptoms attributed to non-refluxing PM. A narrative description is displayed in Appendix 10.

#### Further secondary outcomes

Data on adverse effects, costs of intervention, and health-related quality of life were not reported.

#### Subgroup analyses

Subgroup analyses were not possible due to imprecise reporting of participant characteristics and outcomes or not separable data.

### Sensitivity analyses and publication bias

Sensitivity analyses regarding the outcomes resolution and secondary surgical intervention did not affect the overall pooled prevalence and heterogeneity (Appendices 11 and 12). The associated funnel plots showed asymmetrical distribution of the included studies suggesting a publication bias (Appendices 13 and 14).

## Discussion

In the daily clinical routine, children with non-refluxing PM are currently managed mainly by a non-surgical approach. This trend resulted from observational studies indicating that the majority of these patients can be treated safely without primary surgical intervention [1, 2, 11, 28, 29]. The purpose of this systematic review was to explore whether this approach is based on high-quality and reliable data.

Surprisingly, albeit non-refluxing PM is a relatively common condition, there were only a few studies with small study populations eligible for this systematic review. Furthermore, the primary outcome of interest, change of DRF, was reported insufficiently by the majority of the studies, at least in the follow-up of affected patients. The lack of this important data impedes a reliable statement in determining whether decision-making could be confirmed by the further course of DRF.

Likewise, changes in urinary drainage during follow-up could not be evaluated, due to similar limitations of the reported data. These results are even more astonishing, when considering DRF and urinary drainage as key elements in the decision-making process for surgical intervention in non-refluxing PM [1, 8, 9, 30, 31].

Another important criterion is the grade of ureteral and pelvicalyceal dilatation preferably assessed by ultrasound [1, 8, 9, 30, 31]. While information on persisting or progressive upper urinary tract dilatation was also inconsistent, resolution of PM based on ultrasound findings was reported in 42 to 78% of the PMs. Even though the definition of resolution was often imprecise and inhomogeneous, resolution assessed by ultrasound could be interpreted as a consequence of improved urinary drainage, and therefore a presumed lower risk of DRF deterioration. In general, ultrasound evaluation appeared to be less important by the majority of the included studies, but bias due to underreporting cannot be ruled out. Together with the low pooled prevalence of secondary surgical intervention (13%), the high proportion of resolved PMs seems to support the currently favored non-surgical approach in children with non-refluxing PM.

The lack of consensus guidelines results in various clinical practices for the indication of primary and secondary surgical intervention. Overall, there was a trend to early, primary surgical treatment in symptomatic patients, patients with apparent impaired DRF or a suggested higher risk of deteriorating DRF due to urinary drainage impairment, or extended pelvicalyceal dilatation at initial assessment.

Notwithstanding, the question arises whether some of these patients could have been managed by a primary non-surgical approach. This cautious approach in the decision-making process for surgical intervention is often driven by the limitations of imaging methods to identify patients at risk for DRF worsening [32–34]. Especially within the first weeks of life, impaired DRF is rather a consequence of prenatal than postnatal damage [35]. In addition, based on the available data it is not clear if patients with deteriorating DRF during follow-up may recover after surgical intervention [35]. Finally, the decision of primary surgical intervention in patients with decreased DRF with or without suspected obstruction must be considered a selection bias. When only children with slightly remarkable findings were chosen as candidates for a primary non-surgical approach, the generalizability of the data collected is limited.

Again, the lack of generally accepted clear imaging criteria also applies to children with secondary surgical intervention. In accordance with current consensus criteria, radiological findings including DRF < 40 (− 45)%, decrease in DRF of at least 5–10% in serial functional imaging, and/or progressive dilatation on ultrasound was mostly accepted as indication for surgical intervention in asymptomatic patients [1, 8, 9, 30, 31]. Weighting and interpretation of the functional imaging findings, however, revealed considerable variations, suggesting personal or institutional preferences. Even though current recommendations suggest a combination of different imaging criteria, in many studies, only partial aspects were considered, which leads to a limitation of the already imprecise diagnostic imaging methods. Interestingly, about one-quarter of PMs were managed by secondary surgical intervention based on assumed relevant obstruction only, without adequately considering DRF. Given the ambiguity in interpretation of urinary drainage, this finding is even more questionable.

The majority of patients with non-refluxing PM were diagnosed pre- and postnatally which would have allowed a profound statement on the course of DRF under surveillance. However, observation time was predominantly too short and data following secondary surgical intervention either not available or incomplete to permit an adequate statement about this important aspect. Notwithstanding, the few reported PMs with impaired DRF did not show DRF improvement post-operatively, with missing data on time of follow-up imaging limiting the overall assessment.

In children with symptoms attributed to non-refluxing PM, the indication for surgical intervention is often given more generously [1, 8, 9, 30]. Although early decision-making for surgical intervention can avoid serious clinical consequences, it must be noted critically that the frequency of some symptoms is often comparable to the healthy general population, and proceedings based only on hypothetical explanatory models.

## Limitations

This systematic review was subject to the inherent limitations of the included studies with small sample sizes, mainly monocentric retrospective study design from European centers and moderate to high ROB. Although the literature search was extensive, only a small data set was eligible for this review. A substantial number of studies were excluded due to non-extractable data in mixed study populations or imprecise information regarding follow-up.

The analyzed study population revealed a high clinical heterogeneity including some potential confounders across all studies, such as predominance of PMs with DRF > 40%, contralateral kidney anomalies, not excluded vesicoureteral reflux and ureteral dilatation < 7 mm, leading to a moderate to severe ROB at the participant selection domain.

Another critical aspect is the fact that the majority of the studies included patients with bilateral non-refluxing PM, although bilateral deterioration of kidney function might not be detected by diagnostic imaging, especially if the functional deterioration is of the same extent for both kidneys. Data for this group of patients were separable only partially. However, with the exception of three studies, this bias affects all studies equally to a low percentage (< 20% of included patients).

Though investigations performed in the included studies were relatively uniform, classification systems used and reporting differed [36–41]. This is attributable to methodological limitations, lacking standardized protocols, and conflicting recommendations with respect to result interpretation [1–3, 32–34].

Another barrier was the incongruent reporting of outcome parameters which did not allow appropriate meta-analytical calculations of the majority of the pre-defined subgroup and outcome analyses. Within this context, the development of risk-stratified strategies was not possible.

All these critical issues of high clinical relevance impair the generalizability of the findings and complicate profound counseling and decision-making in children with non-refluxing PM. In summary, the current management strategy needs to be critically scrutinized based on the quality and limitations of the available body of evidence.

## Conclusions

This systematic review emphasizes the need for high-quality and comparable studies to develop more evidence-based recommendations for the current management of children with non-refluxing PM. The critical issues found in this analysis could be overcome by larger, interdisciplinary, well-designed, and prospective studies with standardized imaging protocols, congruent interpretation of the imaging findings, and precise reporting of quantitative outcome data.

Only then can clinical decision-making in terms of non-surgical management and follow-up strategy, patient counseling, and timely surgical intervention to avoid non-restorable deterioration of DRF be facilitated.

### Supplementary information

Below is the link to the electronic supplementary material.

Supplementary file1 (PDF 1545 KB)

## Data Availability

All data relevant to this review are included in the article or uploaded as supplementary information.

## Abbreviations

CI: Confidence interval
DRF: Differential renal function
PM: Primary megaureter
ROB: Risk of bias
^99m^Tc-DMSA: Technetium-99 m-dimercaptosuccinid-acid
^99m^Tc-DTPA: Technetium-99 m-diethylenetriamine-pentaacetic-acid
^99m^Tc-MAG3: Technetium-99 m-mercaptoacetyltriglycine
