# Supplementary material for: Non-surgical management in children with non-refluxing primary megaureter: a systematic review and meta-analysis
Source: Pediatr Nephrol. 2023 Mar 30;38(11):3549–58. doi: 10.1007/s00467-023-05938-6 (PMC10514100; doi:10.1007/s00467-023-05938-6)
Supplement: Supplementary file 1 — Supplementary file1 (PDF 1545 KB) [file 467_2023_5938_MOESM1_ESM.pdf]

Supplementary Information

Non-surgical management in children with non-refluxing primary megaureter: a systematic review and meta-analysis

Authors: Kathrin Buder[1], Kathrin Opherk[1], Sara Mazzi[2], Katharina Rohner[2], Marcus Weitz[1]

Affiliations: [1]University Hospital Tübingen, University Children`s Hospital, Department of General Pediatrics and Hematology/Oncology, Hoppe-Seyler-Str. 1, D – 72076 Tübingen, Germany; [2]University Children`s Hospital Zurich, Pediatric Nephrology Department, Steinwiesstr. 75, CH – 8032 Zurich, Switzerland

Corresponding author: Kathrin Buder; Email: kathrin.buder@med.uni-tuebingen.de

Index

Appendix 1: Electronic search strategy used for identifying of potential studies 3
Appendix 2: Conference proceedings and clinical trials registries 4
Appendix 3: Excluded studies (N=68) 6
Appendix 4: Study characteristics 12
Appendix 5: Definitions and classifications of non-refluxing primary megaureter in the included studies 28
Appendix 6: Characteristics of non-refluxing primary megaureter at enrolment in the included studies 30
Appendix 7: Indications for surgical intervention 33
Appendix 8: Outcome assessment in the included studies 34
Appendix 9: Differential renal function in patients undergoing surgical intervention over time 36
Appendix 10: Symptoms in non-refluxing primary megaureter 38
Appendix 11: Sensitivity analyses regarding the pooled prevalence of resolution in non-refluxing primary megaureter 39
Figure 11.1: Studies excluding renal units with suspected obstruction or with missing information on urinary drainage 39
Figure 11.2: Studies including all types of non-refluxing primary megaureter in terms of urinary drainage (studies without precise reporting of this data excluded) 39
Figure 11.3: Studies with <30 renal units 39
Figure 11.4: Studies with ≥50 renal units 39
Figure 11.5: Studies published beyond 2000 40
Figure 11.6: Studies with follow-up ≥12 months 40
Appendix 12: Sensitivity analyses regarding the pooled prevalence of secondary surgery in non-refluxing primary megaureter 41
Figure 12.1: Studies excluding renal units with suspected obstruction or with missing information on urinary drainage 41
Figure 12.2: Studies including all types of non-refluxing primary megaureter in terms of urinary drainage (studies without precise reporting of this data excluded) 41
Figure 12.3: Studies with <30 renal units 41
Figure 12.4: Studies with ≥50 renal units 41
Figure 12.5: Studies published beyond 2000 42
Figure 12.6: Studies including follow-up <12 months 42
Figure 12.7: Studies with follow-up ≥12 months 42
Appendix 13: Funnel plot regarding the outcome resolution 43
Appendix 14: Funnel plot regarding the outcome secondary surgery 44
References 45

Appendix 1: Electronic search strategy used for identifying of potential studies

Database	#	Search terms
CENTRAL (Cochrane Central Register of Controlled Trials)	1	(megaureter* OR "mega-ureter*" OR megadolichoureter* OR "megalo ureter*" OR megaloureter* OR VUJO OR UVJO OR "vesico-ureter*" OR vesicoureter* OR ureterovesic* OR "uretero-vesic*" OR ((ureter* OR junction* OR UT) NEAR/1 (dilat* OR obstruct*))):ti,ab,kw
	2	(((hydronephro* OR hydroureteronophro*) NEAR/5 (prenatal OR neonatal OR antenatal OR congenital))):ti,ab,kw
	3	#1 OR #2
MEDLINE (Ovid)	1	exp Ureteral Obstruction/ or exp Vesico-Ureteral Reflux/or megaureter*.ti,ab. or mega-ureter*.ti,ab. or megadolichoureter*.ti,ab. or megalo ureter*.ti,ab. or megaloureter*.ti,ab. or VUJO.ti,ab. or UVJO.ti,ab. or vesico-ureter*.ti,ab. or vesicoureter*.ti,ab. or ureterovesic*.ti,ab. or uretero-vesic*.ti,ab. or ((ureter* or junction* or UT) adj1 (dilat* or obstruct*)).ti,ab.
	2	((hydronephro* or hydroureteronophro*) adj5 (prenatal or neonatal or antenatal or congenital)).ti,ab. or (Hydronephrosis/ and (prenatal or neonatal or antenatal).ti,ab.)
	3	1 or 2
	4	3 not (animals not humans).sh.
	5	4 not (exp adult/ not (exp child/ or exp infant/ or exp adolescent/))
EMBASE (Embase)	1	'megaureter'/exp OR megaureter*:ti,ab OR 'mega-ureter*':ti,ab OR megadolichoureter*:ti,ab OR 'megalo ureter*':ti,ab OR megaloureter*:ti,ab OR vujo:ti,ab OR uvjo:ti,ab OR 'vesico-ureter*':ti,ab OR vesicoureter*:ti,ab OR ureterovesic*:ti,ab OR 'uretero-vesic*':ti,ab OR (((ureter* OR junction* OR ut) NEAR/1 (dilat* OR obstruct*)):ti,ab)
	2	(((hydronephro* OR hydroureteronophro*) NEAR/5 (prenatal OR neonatal OR antenatal OR congenital)):ti,ab) OR ('hydronephrosis'/exp AND (prenatal:ti,ab OR neonatal:ti,ab OR antenatal:ti,ab))
	3	#1 OR #2
	4	#3 NOT (([animals]/lim OR 'nonhuman'/exp) NOT [humans]/lim)
	5	#4 NOT ('adult'/exp NOT 'juvenile'/exp)

Appendix 2: Conference proceedings and clinical trials registries

American Academy of Pediatrics (AAP)<br>Conference: AAP Experience (annual)
2017: https://publications.aap.org/pediatrics/issue/142/1_MeetingAbstract<br>2018: https://publications.aap.org/pediatrics/issue/144/2_MeetingAbstract<br>2019: https://publications.aap.org/pediatrics/issue/146/1_MeetingAbstract<br>2020: https://publications.aap.org/pediatrics/issue/147/3_MeetingAbstract<br>2021: https://publications.aap.org/pediatrics/issue/149/1%20Meeting%20Abstracts%20February%202022<br>2022: https://eventscribe.net/2022/AAPexperience/searchbyposterbucket.asp?f=PosterSessionName&pfp PosterSessionName
European Society for Pediatric Urology (ESPU)<br>Conference: Congress of the ESPU
2017 – 2022: https://www.espu.org/members/previous-events-abstracts
Society for Pediatric Urology (SPU)<br>Conferences: SPU Annual Meeting; Pediatric Urology Fall Congress
2017 – 2022: Annual meeting: https://spuonline.org/meeting/Archives<br>2017 – 2022:[1] Fall Congress: http://spuonline.org/fallcongress/Archives/<br>[1]Data about fall Congress of year 2020 not available
American Urological Association (AUA)<br>Conference: AUA Annual Meeting
2017: https://www.jurology.com/issue/S0022-5347(17)X0003-7<br>2018: https://www.auajournals.org/issue/S0022-5347(18)X0002-0<br>2019: https://www.auajournals.org/toc/juro/201/Supplement+4?pageStart=18&pageSize=100<br>2020: https://www.auajournals.org/toc/juro/203/Supplement+4<br>2021: https://www.auajournals.org/toc/juro/206/Supplement+3<br>2022: https://www.auajournals.org/toc/juro/207/Supplement+5
European Association of Urology (EAU)<br>Conference: Annual EAU Conference
2017: https://www.sciencedirect.com/journal/european-urology-supplements/vol/16/issue/3<br>2018: https://resource-centre.uroweb.org/resource-centre/eau18<br>2019: https://resource-centre.uroweb.org/resource-centre/eau19<br>2020: https://resource-centre.uroweb.org/resource-centre/eau20v<br>2021: https://virtual.uroweb.org/resource-centre/EAU21<br>2022: https://resource-centre.uroweb.org/resource-centre/eau22
European Academy of Pediatrics (EAP)<br>Conference: EAP Congress
2017: https://link.springer.com/content/pdf/10.1007%2Fs00431-017-2979-8.pdf<br>2018: https://eaps.kenes.com/2018/scientific-information/scientific-programme#.WkOj4FWWaUk<br>2019: https://link.springer.com/content/pdf/10.1007/s00431-019-03466-w.pdf<br>2020: https://eaps2020.kenes.com/wp-content/uploads/sites/43/2020/10/EAPS-2020-programme-2.pdf<br>2021: not available<br>2022: https://eaps2022.kenes.com/scientific-programme/
American Society of Pediatric Nephrology (ASPN)<br>Conference: Pediatric Academic Societies' Annual Meeting
2017: https://www.xcdsystem.com/pas/program/2017/index.cfm?pgid=156&RunRemoveSessionFilter=1<br>2018: https://www.xcdsystem.com/pas/program/2018/index.cfm?pgid=156<br>2019 – 2022: not available

Society for Pediatric Nephrology (ESPN)<br>Annual meeting
2017: https://link.springer.com/article/10.1007/s00467-017-3753-x<br>2018: https://www.espn2018.org/scientific.programme.php<br>2019: not available<br>2020: https://www.espn-online.org/wp-content/uploads/2020/10/espn-2020-programme.pdf<br>2021: https://link.springer.com/content/pdf/10.1007/s00467-021-05210-9.pdf<br>2022: https://link.springer.com/content/pdf/10.1007/s00467-022-05630-1.pdf
**Clinical trials registries**
• Clinical trials: https://www.clinicaltrials.gov<br>• ISRCTN registry: https://www.isrctn.com/<br>• Trials Central: https://www.cochranelibrary.com/central/about-central<br>• World Health Organization (WHO) International Clinical Trials Registry Platform (ICTRP): https://trialsearch.who.int/<br>• Internet Portal of the German Clinical Trials Register (DRKS): https://www.bfarm.de/DE/Das-BfArM/Aufgaben/Deutsches-Register-Klinischer-Studien/_node.html<br>• Australian New Zealand Clinical Trials Registry (ANZCTR): https://www.anzctr.org.au/trialSearch.aspx

Appendix 3: Excluded studies (N=68)

Study	Reason for exclusion
Anheuser P, Kranz J, Steffens J, Beetz R (2013) [Primary megaureter]. Urologe A 52:33-38.	Review article
Arena S, Magno C, Montalto AS, Russo T, Mami C, Baldari S, Romeo C, Arena F (2012) Long-term follow-up of neonatally diagnosed primary megaureter: rate and predictors of spontaneous resolution. Scand J Urol Nephrol 46:201-207.	Several outcome data regarding study objective not separable
Ardissino G, Dacco V, Testa S, Bonaudo R, Claris-Appiani A, Taioli E, Marra G, Edefonti A, Sereni F, ItalKid P (2003) Epidemiology of chronic renal failure in children: data from the ItalKid project. Pediatrics 111:e382-387.	Not study objective
Areses Trapote R, Urbieta Garagorri MA, Ubetagoyena Arrieta M, Arruebarrena Lizarraga D, Alzueta Beneite MT, Eizaguirre Sexmilo I, Rodriguez Mazorriaga F, Esparza Paz P, Emparanza Knorr JI (2006) [Severe primary congenital unilateral hydronephrosis. A review of 98 cases]. Anales de Pediatria 64:11-20.	Several outcome data regarding study objective not separable
Avni EF, Pichot E, Schulman CC (1992) Neonatal congenital megaureters: trends in diagnosis and management. World Journal of Urology 10:90-93.	Ineligible outcomes of interest reported
Babut JM, Fremond B, Sameh A, Vidal V (1988) Primary megaureter in the neonate with prenatal or postnatal diagnosis. Zeitschrift fur Kinderchirurgie 43:150-153.	Mixed study population including associated ipsilateral urinary tract anomalies; ineligible outcomes of interest reported
Baskin LS, Zderic SA, Snyder HM, Duckett JW (1994) Primary dilated megaureter: long-term followup. J Urol 152:618-621.	Imprecise outcomes regarding study objective
Braga LH, D'Cruz J, Rickard M, Jegatheeswaran K, Lorenzo AJ (2016) The Fate of Primary Nonrefluxing Megaureter: A Prospective Outcome Analysis of the Rate of Urinary Tract Infections, Surgical Indications and Time to Resolution. Journal of Urology 195:1300-1305.	Several outcome data regarding study objective not separable
Brueziere J (1974) [Primary mega-ureter in infants. Etiologic, anatomic and therapeutic studies apropos of 100 cases in 71 children]. Chirurgie 100:712-723	Mixed study population; ineligible outcomes of interest reported
Chertin B, Pollack A, Koulikov D, Rabinowitz R, Shen O, Hain D, Hadas-Halpren I, Shenfeld OZ, Farkas A (2008) Long-term follow up of antenatally diagnosed megaureters. J Pediatr Urol 4:188-191.	Several outcome data regarding study objective not separable
Cox R, Strachan JR, Woodhouse CR (1990) Twenty-year follow-up of primary mega-ureter. European Urology 17:43-46.	Several outcome data regarding study objective not separable
Cozzi F, Madonna L, Maggi E, Piacenti S, Bonanni M, Roggini M, Capocaccia P, Pachi A (1993) Management of primary megaureter in infancy. Journal of Pediatric Surgery 28:1031-1033.	Several outcome data regarding study objective not separable

Davenport MT, Merguerian PA, Koyle M (2013) Antenatally diagnosed hydronephrosis: current postnatal management. Pediatr Surg Int 29:207-214.	Review article
de Jong TP (1997) Treatment of the neonatal and infant megaureter in reflux, obstruction and complex congenital anomalies. Acta Urologica Belgica 65:45-47.	Ineligible outcomes of interest reported
Dekirmendjian A, Braga LH (2019) Primary Non-refluxing Megaureter: Analysis of Risk Factors for Spontaneous Resolution and Surgical Intervention. Frontiers in Pediatrics 7:126.	Several data (unilateral versus bilateral primary megaureters, primary versus secondary surgery) not separable
DiRenzo D, Persico A, DiNicola M, Silvaroli S, Martino G, LelliChiesa P (2015) Conservative management of primary non-refluxing megaureter during the first year of life: A longitudinal observational study. J Pediatr Urol 11:226 e221-226.	Follow-up data for only 12 months and therefore ineligible for study objective
Domini M, Aquino A, Pappalepore N, Tursini S, Marino N, Strocchi F, Lelli Chiesa P (1999) Conservative treatment of neonatal primary megaureter. European Journal of Pediatric Surgery 9:396-399.	Imprecise outcomes regarding study objective
Drlik M, Flogelova H, Martin K, Jan T, Pavel Z, Oldrich S, Ivo N, Martin K, Radim K (2016) Isolated low initial differential renal function in patients with primary non-refluxing megaureter should not be considered an indication for early surgery: A multicentric study. Journal of pediatric urology 12:231.e231-234.	Several outcome data (particularly primary and secondary surgical intervention) regarding study objective not separable
Ghanmi S, Ben Hamouda H, Krichene I, Soua H, Ayadi A, Souissi MM, Hamza H, Nouri A, Essabbeh H, Sfar MT (2011) [Management and follow-up of antenatally diagnosed primary megaureters]. Progres en Urologie 21:486-491.	Mixed study population; outcome data not separable
Gomez Fraile A, Anton-Pacheco J, Aransay Bramtot A, Lopez Vazquez F, Serrano C, Manrique A (1993) [Clinical course of primary non-obstructive megaureter: analysis of our series]. Actas Urologicas Espanolas 17:351-356.	Study population published also in a further publication; the latter included in this review:<br>Anton-Pacheco Sanchez J, Gomez Fraile A, Aransay Brantot A, Lopez Vazquez F, Encinas Goenechea A (1995) Diuresis renography in the diagnosis and follow-up of nonobstructive primary megaureter. Eur J Pediatr Surg 5:338-341.
Farrugia MK, Hitchcock R, Radford A, Burki T, Robb A, Murphy F, British Association of Paediatric U (2014) British Association of Paediatric Urologists consensus statement on the management of the primary obstructive megaureter. J Pediatr Urol 10:26-33.	Review article
Hamid R, Bhat NA, Baba AA, Mufti GN, Sheikh KA, Bashir MI (2022) Primary obstructive megaureter in children; 10 years' experience from a tertiary care center. Urol Ann 14:252-258.	Several data (unilateral versus bilateral megaureters) not separable
Hanna MK, Jeffs RD (1975) Primary obstructive megaureter in children. Urology 6:419-427.	Ineligible outcomes of interest reported

Herz D, Merguerian P, McQuiston L (2014) Continuous antibiotic prophylaxis reduces the risk of febrile UTI in children with asymptomatic antenatal hydronephrosis with either ureteral dilation, high-grade vesicoureteral reflux, or ureterovesical junction obstruction. J Pediatr Urol 10:650-654.	Not study objective
Hodges SJ, Werle D, McLorie G, Atala A (2010) Megaureter. ScientificWorldJournal 10:603-612.	Review article
Hollard D, Schmitt M, Prevot J (1982) Primitive megalo-ureters in children. 52 observations. Annales Medicales de Nancy et de l'Est 21:487-491.	Ineligible outcomes of interest reported
Hoquetis L, Le Mandat A, Bouali O, Ballouhey Q, Mouttalib S, Moscovici J, Galinier P (2013) [Primary obstructive megaureters: long-term follow-up]. Progres en Urologie 23:470-473.	Mixed study population; data not separable; ineligible outcomes of interest reported
Keating MA (1990) A different perspective of the perinatal primary megaureter. Problems in Urology 4:583-594.	Several outcome data (particularly differential renal function) regarding study objective not reported
Lee JH, Choi HS, Kim JK, Won HS, Kim KS, Moon DH, Cho KS, Park YS (2008) Nonrefluxing neonatal hydronephrosis and the risk of urinary tract infection. J Urol 179:1524-1528.	Mixed study population; data not separable; ineligible outcomes of interest reported
Maizels M, Reisman ME, Flom LS, Nelson J, Fernbach S, Firlit CF, Conway JJ (1992) Grading nephroureteral dilatation detected in the first year of life: correlation with obstruction. Journal of Urology 148:609-614; discussion 615-606.	Mixed study population; data not separable; ineligible outcomes of interest reported
Martin JA, Piero JL, Piro C, Chicaiza E, Gosalbez R (1998) [Ten years of prenatal diagnosis of uropathies. Study and conclusions]. Cirugia Pediatrica 11:55-63.	Ineligible outcomes of interest reported
McLellan DL, Retik AB, Bauer SB, Diamond DA, Atala A, Mandell J, Lebowitz RL, Borer JG, Peters CA (2002) Rate and predictors of spontaneous resolution of prenatally diagnosed primary nonrefluxing megaureter. J Urol 168:2177-2180; discussion 2180.	Data (unilateral versus bilateral megaureters) not separable
Merlini E, Rotundi F, Seymandi P, Santini L (2002) [Primary megaureter detected during the first year of life. Review of case reports in the last 10 years and analysis of prognostic factors]. Pediatria Medica e Chirurgica 24:220-222.	Ineligible outcomes of interest reported
Meyer JS, Lebowitz RL (1992) Primary megaureter in infants and children: a review. Urol Radiol 14:296-305.	Ineligible outcomes of interest reported
Mollard P, Paillot JM (1973) Primary megaureter (pathogenesis and treatment 104 patients—131 ureters). Prog Pediatr Surg 5:113-134.	Ineligible outcomes of interest reported
Mollard P, Foray P, De Godoy JL, Valignat C (1993) Management of primary obstructive megaureter without reflux in neonates. European Urology 24:505-510.	Ineligible outcomes of interest reported

Mollard P, Bonnet JP, Vautherin R (1995) Primary obstructive megaureter without reflux in neonates. Annales de Pediatrie 42:249-255.	Ineligible outcomes of interest reported
Nagy V, Baca M, Boor A (2013) Primary obstructed megaureter (POM) in children. Bratisl Lek Listy 114:650-656.	Several outcome data (particularly primary versus secondary surgical intervention, unilateral versus bilateral megaureters) not separable, furthermore data on functional imaging at enrolment missing in a high proportion of patients
Nguyen HT, Herndon CD, Cooper C, Gatti J, Kirsch A, Kokorowski P, Lee R, Perez-Brayfield M, Metcalfe P, Yerkes E, Cendron M, Campbell JB (2010) The Society for Fetal Urology consensus statement on the evaluation and management of antenatal hydronephrosis. J Pediatr Urol 6:212-231.	Review article
Peters CA, Mandell J, Lebowitz RL, Colodny AH, Bauer SB, Hendren WH, Retik AB (1989) Congenital obstructed megaureters in early infancy: diagnosis and treatment. Journal of Urology 142:641-645; discussion 667-648.	Not study objective
Perdzynski W, Kalicinski ZH (1996) Long-term results after megaureter folding in children. J Pediatr Surg 31:1211-1217.	Not study objective
Picart B, Pons M, Line A, Francois C, Poli Merol ML (2017) [Therapeutic mega-ureter primitive before one year of life, retrospective study of 20years]. Progres en Urologie 27:103-109.	Mixed study population; data regarding study objective not separable
Pitts WR, Jr., Muecke EC (1974) Congenital megaloureter: a review of 80 patients. J Urol 111:468-473.	Ineligible outcomes of interest reported
Pourvaziri A, Amjad G, Mehdizadeh M (2016) Drainage related ultra sonography; acute us technique in categorizing primary mega ureter. Acta Medica Mediterranea 32:2121-2126.	Not study objective
Rabinowitz R, Barkin M, Schillinger JF, Jeffs RD (1979) Surgical treatment of the massively dilated primary megaureter in children. Br J Urol 51:19-23.	Not study objective
Ranawaka R, Hennayake S (2013) Resolution of primary non-refluxing megaureter: an observational study. Journal of Pediatric Surgery 48:380-383.	Ineligible outcomes of interest reported (particularly no information on differential renal function)
Renda R (2018) Renal outcome of congenital anomalies of the kidney and urinary tract system: a single-center retrospective study. Minerva Urol Nefrol 70:218-225.	Mixed study population; data not separable; ineligible outcomes of interest reported
Rickwood AM, Jee LD, Williams MP, Anderson PA (1992) Natural history of obstructed and pseudo-obstructed megaureters detected by prenatal ultrasonography. British Journal of Urology 70:322-325.	Several outcome data (unilateral versus bilateral primary megaureters) not separable

Ring E, Petritsch P, Riccabona M, Vilits P, Haim-Kuttnig M, Hubmer G (1992) [Prenatal diagnosis of primary megaureter]. Klinische Padiatrie 204:382-385.	Mixed study population with ipsilateral urinary tract anomalies, data not separable, ineligible outcomes of interest reported
Roth CC, Hubanks JM, Bright BC, Heinlen JE, Donovan BO, Kropp BP, Frimberger D (2009) Occurrence of urinary tract infection in children with significant upper urinary tract obstruction. Urology 73:74-78.	Not study objective
Rubenwolf P, Herrmann-Nuber J, Schreckenberger M, Stein R, Beetz R (2016) Primary non-refluxive megaureter in children: single-center experience and follow-up of 212 patients. International Urology & Nephrology 48:1743-1749.	Ineligible outcomes of interest reported (particularly due to functional imaging not routinely performed)
Savanelli A, Baltogiannis D, De Lucia A, Errico D, Sordino D, Tilemis S, Settimi A, Rambaldi P, Dolezalova H, Vallone G (2006) [Current trend in the diagnosis and treatment of primary non refluxing megaureter]. Pediatria Medica e Chirurgica 28:95-100.	Several outcome data regarding study objective not reported or not separable
Sanna-Cherchi S, Ravani P, Corbani V, Parodi S, Haupt R, Piaggio G, Innocenti ML, Somenzi D, Trivelli A, Caridi G, Izzi C, Scolari F, Mattioli G, Allegri L, Ghiggeri GM (2009) Renal outcome in patients with congenital anomalies of the kidney and urinary tract. Kidney Int 76:528-533.	Not study objective
Shamshirsaz AA, Ravangard SF, Egan JF, Prabulos AM, Shamshirsaz AA, Ferrer FA, Makari JH, Leftwich HK, Herbst KW, Billstrom RA, Sadowski A, Gurram P, Campbell WA (2012) Fetal hydronephrosis as a predictor of neonatal urologic outcomes. Journal of Ultrasound in Medicine 31:947-954.	Mixed study population; only 2 megaureters included; ineligible outcomes of interest reported
Shokeir AA, Nijman RJ (2000) Primary megaureter: current trends in diagnosis and treatment. BJU Int 86:861-868.	Review article
Shukla AR, Cooper J, Patel RP, Carr MC, Canning DA, Zderic SA, Snyder HM, 3rd (2005) Prenatally detected primary megaureter: a role for extended followup. J Urol 173:1353-1356.	Detailed data on functional imaging not reported; therefore, ineligible outcomes of interest
Signorelli M, Cerri V, Taddei F, Groli C, Bianchi UA (2005) Prenatal diagnosis and management of mild fetal pyelectasis: implications for neonatal outcome and follow-up. European Journal of Obstetrics, Gynecology, & Reproductive Biology 118:154-159.	Mixed study population; ineligible outcomes of interest reported
Sinha A, Bagga A, Krishna A, Bajpai M, Srinivas M, Uppal R, Agarwal I (2013) Revised guidelines on management of antenatal hydronephrosis. Indian J Nephrol 23:83-97.	Review article
Song SH, Lee SB, Park YS, Kim KS (2007) Is antibiotic prophylaxis necessary in infants with obstructive hydronephrosis? J Urol 177:1098-1101; discussion 1101.	Not study objective

Tröbs RB, Heinecke K, Elouahidi T, Nounla J, Kluge R (2006) Renal function and urine drainage after conservative or operative treatment of primary (obstructive) megaureter in infants and children. International Urology & Nephrology 38:141-147.	Several outcome data (particularly primary versus secondary surgery) not separable
Unger G, Finke G (1979) [Results of follow-up studies in children with megaureters]. Zeitschrift fur Urologie und Nephrologie 72:375-381.	Not study objective
Valent-Moric B, Zigman T, Cuk M, Zaja-Franulovic O, Malenica M (2011) Postnatal evaluation and outcome of infants with antenatal hydronephrosis. Acta Clinica Croatica 50:451-455.	Not study objective
Vereecken RL, Proesmans W (1999) A review of ninety-two obstructive megaureters in children. European Urology 36:342-347.	Ineligible outcomes of interest reported
Vidal V, Fremond B, Chapuis M, Babut JM (1988) [Primary obstructive megaureter in infants: medical or surgical treatment? Apropos of 24 cases]. J Urol (Paris) 94:279-283.	Not study objective
Wilcox D, Mouriquand P (1998) Management of megaureter in children. Eur Urol 34:73-78.	Review article
Woodward M, Frank D (2002) Postnatal management of antenatal hydronephrosis. BJU Int 89:149-156.	Review article
Zampieri N, Zamboni C, Camoglio FS (2011) Clinical course of grade I-III megaureters detected on prenatal ultrasound. Minerva Pediatrica 63:439-443.	Several outcome data not separable
Zareba P, Lorenzo AJ, Braga LH (2014) Risk factors for febrile urinary tract infection in infants with prenatal hydronephrosis: comprehensive single center analysis. Journal of Urology 191:1614-1618.	Not study objective

Appendix 4: Study characteristics

**Antón-Pacheco Sanchez (1995)** [1]	
**Methods**	
Study design	Retrospective cohort study
Country	Spain
Setting	Monocentric, tertiary center
Power analysis	Not reported
Funding sources	Not reported
Conflict of interest	Not reported
Study dates	1981-1992
**Participants – INCLUSION and EXCLUSION CRITERIA, COMORBIDITIES, POTENTIAL CONFOUNDERS**	
Inclusion criteria	Diagnosis of primary non-obstructed megaureter
Exclusion criteria	Obstructive urinary drainage pattern on functional imaging
Comorbidities	1 patient: contralateral renal agenesis<br>2 patients: contralateral VUR II°<br>1 patient: craniosynostosis
Confounders	1 patient: contralateral renal agenesis<br>2 patients: contralateral VUR II°<br>Bilateral cases
**Definitions**	
Megaureter in terms of ureteral dilatation (mm)	Not reported
Megaureter in terms of IVP	Grading due to Pfister Hendren classification [2]
Pelvicalyceal dilatation	Grading due to IVP using Pfister Hendren classification [2]
Urinary drainage (functional imaging)	Grading based on O`Reilly criteria [3, 4] and clearance t ½<br><u>Curve type:</u><br>• Curve type I: normal<br>• Curve type II: obstructed<br>• Curve type IIIa: dilated without obstruction<br>• Curve type IIIb: dilated and partially obstructed<br><u>Clearance t½:</u><br>• <15 min: non-obstructed<br>• 15-20 min: partially obstructed<br>• >20 min: obstructed
DRF (functional imaging)	• DRF <30%: poor renal function<br>• DRF 30-40%: moderate renal function<br>• DRF >40%: good renal function
Obstruction	Defined according to O`Reilly criteria / drainage pattern and clearance t½:<br>• Curve type II
Resolution	Not precisely defined; normalization of US and urinary drainage pattern / renogram reported
**Participants – GROUP CHARACTERISTICS**	
Total number of participants, n	23
Number of renal units, n (total / left / right / bilateral)	26 / 13 / 7 / 3
Female, n (%)	7 (30%)
Age at diagnosis of PM	16 patients prenatally<br>7 patients postnatally, range 3-108 months
Mean / median age of study participants (range)	Not reported
Ureteral dilatation, mm	Not reported
Ureteral dilatation, assessment by IVP	Pfister-Hendren classification:<br>• Moderate: 22 renal units<br>• Ureteral dilatation not precisely reported: 4 renal units
Pelvicalyceal dilatation	Reported, but not graded: 13 left, 7 right, 3 bilateral renal units (in total: 16 renal units)

Urinary drainage and DRF	• IIIa, DRF >40%; t½ <15 min: 13 renal units<br>• IIIa, DRF >40%; t½ 15-20 min: 5 renal units<br>• IIIa, DRF 30-40%; t½ <15 min: 1 renal unit<br>• IIIb, DRF >40%; %; t½ <15 min: 6 renal units<br>• IIIb, DRF 30-40%; t½ <15 min: 1 renal unit
Follow-up duration, months	12-96
Loss of follow-up, n	Not reported
**Interventions**	
Planned interventions: 1) at PM diagnosis: US, VCUG, ^{99m}Tc-DTPA or ^{99m}Tc-MAG3 renal scintigraphy, IVP; 2) first year: US + ^{99m}Tc-DTPA or ^{99m}Tc-MAG3 renal scintigraphy every 3 months; in addition IVP in cases with anatomical deterioration in US; 3) if no deterioration: US + ^{99m}Tc-DTPA or ^{99m}Tc-MAG3 renal scintigraphy 1 x / year	
VUR exclusion, procedure	All diseased renal units; VCUG
Functional imaging (type)	^{99m}Tc-DTPA or ^{99m}Tc-MAG3 renal scintigraphy
Furosemide application for functional imaging	Probably, not declared
Bladder drainage during functional imaging	Not reported
Initial threshold for primary surgical intervention	Not reported
Co-interventions	Continuous antibiotic prophylaxis in all patients<br>Regular urine culture assessment
Primary surgical intervention, n (%)	0 (0%)
Temporary diversions	Not reported
**Outcomes** (n=23 patients / n=26 renal units)	
Timing of last assessment	Variable
US findings	• Normal image: 7 renal units<br>• Ureteral dilatation: 2 renal units<br>• Mild-moderate pelvicalyceal-dilatation: 17 renal units<br>• Improvement not precisely reported<br>• Deterioration not precisely reported
Urinary drainage and DRF	• IIIa, DRF ≥40%; t½ <15 min: 17 renal units<br>• I, DRF ≥40%; t½ <15 min: 8 renal units<br>• II, DRF ≥40%; t½ 15-20 min: 1 renal unit
Threshold for secondary surgery	• Not clearly defined<br>• As indicated by the study: symptoms and change to obstructive urinary drainage pattern
Secondary surgical intervention, n (%)	1 renal unit (4%)
Mode of surgery	Not reported
Timing of secondary surgery	12 months after diagnosis
UTI	2 patients
Other clinical complications	Vomiting, hematuria, abdominal pain in occasionally diagnosed cases; data not separable for patients / renal units
Notes	
Potential confounders not excluded	

Abbreviations: DRF, differential renal function; IVP, intravenous pyelogram; PM, primary megaureter; ^{99m}Tc-DTPA, Technetium-99m-diethylenetriamine-pentaacetic-acid; ^{99m}Tc-MAG3, Technetium-99m-mercaptoacetyltriglycine; US, ultrasound; UTI, urinary tract infections; VCUG, voiding cystourethrogram; VUR, vesicoureteral reflux

Arena (1998) [5]	
**Methods**	
Study design	Prospective cohort study
Country	Italy
Setting	Monocentric, tertiary center
Power analysis	Not reported
Funding sources	Not reported
Conflict of interest	Not reported
Study dates	01/1992-12/1994
**Participants – INCLUSION and EXCLUSION CRITERIA, COMORBIDITIES, POTENTIAL CONFOUNDERS**	
Inclusion criteria	Diagnosis of primary non-refluxing neonatal megaureter
Exclusion criteria	Ureteral dilatation not associated with VUR or cervico-ureteral obstruction
Comorbidities	4 patients: contralateral multicystic kidney dysplasia<br>1 patient: contralateral kidney agenesis<br>2 patients: contralateral VUR II°<br>1 patient: pluridysmorphic
Confounders	4 patients: contralateral multicystic kidney dysplasia<br>1 patient: contralateral kidney agenesis<br>2 patients: contralateral VUR II°<br>Bilateral cases
**Definitions**	
Megaureter in terms of ureteral dilatation (mm)	Not reported
Megaureter in terms of IVP	Grading due to Beurton classification [6]
Pelvicalyceal dilatation	Grading due to IVP using Beurton classification [6]
Urinary drainage (functional imaging)	Diuretic test:<br>• Positive: not obstructed<br>• Equivocal<br>• Negative: "obstructed" = accumulation of tracer > 50% 20 min after furosemide application
DRF (functional imaging)	DRF ≤40%: impaired
Obstruction	Due to urinary drainage pattern; see above
Resolution	Not precisely defined; regression in dilatation
**Participants – GROUP CHARACTERISTICS**	
Total number of participants, n	22
Number of renal units, n (total / left / right / bilateral)	24 / 13 / 7 / 2
Female, n (%)	8 (36%)
Age at diagnosis of PM	18 patients prenatal; 4 patients during neonatal screening
Mean / median age of study participants (range)	Not reported
Ureteral dilatation, mm	Not reported
Ureteral dilatation, assessment by IVP	Beurton classification:<br>• Type I: 2 renal units<br>• Type II: 9 renal units<br>• Type III: 13 renal units
Pelvicalyceal dilatation	2 significant (type III), not clearly reported for remaining
Urinary drainage	Diuretic test:<br>• Non-obstructed: 19 renal units<br>• Equivocal: 2 renal units<br>• Obstructed: 2 renal units
DRF	DRF >40%: 23 renal units<br>(1 patient / renal unit not examined [death])
Follow-up duration, months	Median 36, mean 29, range 6-54
Loss of follow-up, n	1 patient (died)

**Interventions**	
Planned interventions: 1) US 2 months in the first year; afterwards every 6 months; 2) ^{99m}Tc-DTPA scintigraphy every 6 months in the first year; afterwards yearly	
VUR exclusion; procedure	All diseased renal units; VCUG
Functional imaging (type)	^{99m}Tc-DTPA renal scintigraphy
Furosemide application for functional imaging	Yes
Bladder drainage during functional imaging	Not reported
Initial threshold for primary surgical intervention	• Not precisely reported<br>• As indicated by the study: severe ureteral and pelvicalyceal dilatation in solitary functioning kidney
Co-interventions	Continuous antibiotic prophylaxis in all patients<br>Regular urine culture assessment
Primary surgical intervention, n (%)	1/24 renal units (4%) nephrostomy (patient died 3 after birth; no assessment regarding obstruction / impaired urinary drainage)
Temporary diversions	1 nephrostomy (patient died 3 after birth)
**Outcomes** (n=21 patients / n=23 renal units; 1 patient / renal unit due to primary surgery excluded; furthermore, outcome parameters with missing data for some patients / renal units)	
Timing of last assessment	Variable
US findings	• Regression/resolution: 12 renal units<br>• Unchanged: 4 renal units<br>• Improvement: 7 renal units<br>• Deterioration: 0 renal units<br>→ classification not defined in detail
Urinary drainage	Not reported (only examples for some cases)
DRF	DRF >40%: 18/23 renal units<br>(5/23 renal units not examined)
Threshold for secondary surgery	• Not clearly defined<br>• As indicated by the study:<br>- Obstructive urinary drainage pattern<br>- Equivocal urinary drainage pattern + persisting PM type III dilatation<br>- Recurrent UTI + PM type III dilatation (with improved urinary drainage pattern) in solitary kidney
Secondary surgical intervention, n (%)	4/23 renal units (17%)
Mode of surgery	Not reported
Timing of secondary surgery	Not uniformly reported: at the age of 6, 12, 30, 36 versus 6, 12, 18 and 24 months
UTI	3 patients
Other clinical complications	1 patient: acute renal failure leading to death
Notes	
Potential confounders not excluded; some data not clearly reported; grading systems and outcome parameters not clearly reported	

Abbreviations: DRF, differential renal function; IVP, intravenous pyelogram; PM, primary megaureter; ^{99m}Tc-DTPA, Technetium-99m-diethylenetriamine-pentaacetic-acid; US, ultrasound; UTI, urinary tract infections; VCUG, voiding cystourethrogram; VUR, vesicoureteral reflux

**Calisti (2008)** [7]	
**Methods**	
Study design	Retrospective cohort study
Country	Italy
Setting	Monocentric, tertiary center
Power analysis	Not reported
Funding sources	Not reported
Conflict of interest	Not reported
Study dates	1991-2006
**Participants – INCLUSION and EXCLUSION CRITERIA, COMORBIDITIES, POTENTIAL CONFOUNDERS**	
Inclusion criteria	Prenatally diagnosed primary megaureter with distal ureteral diagnosis ≥10 mm
Exclusion criteria	VUR, bladder outlet obstruction, urinary tract duplication
Comorbidities	Not reported
Confounders	Not precisely reported<br>Regarding selection of participants: not all patients of the initial cohort included<br>Bilateral cases
**Definitions**	
Megaureter in terms of ureteral dilatation (mm)	≥10 mm
Megaureter in terms of IVP	Grading due to Pfister Hendren classification [2]
Pelvicalyceal dilatation	Grading due to SFU classification [8]; grades 1-5
Urinary drainage (functional imaging)	Not reported
DRF (functional imaging)	DRF interpretation not reported; due to pooling only early phase of ^{99m}Tc-MAG3 parenchymal uptake considered
Obstruction	Not reported
Resolution	Not precisely defined, resolution of dilatation
**Participants – GROUP CHARACTERISTICS**	
Total number of participants, n	37
Number of renal units, n (total / left / right / bilateral)	50 / left and right not reported / 13 bilateral
Female, n (%)	3 (8%)
Age at diagnosis of PM	Prenatal diagnosis, referred within first month after birth
Mean / median age of study participants (range)	Not reported
Ureteral dilatation, mm	Data reported separately for:<br>• Non-surgically treated group (46/50 renal units): mean ureteral diameter: 10.9 mm<br>• Secondary surgically treated group (4/50 renal units): mean ureteral diameter 14.7 mm
Ureteral dilatation, assessment by IVP	Pfister-Hendren classification:<br>• Type I: 16 renal units<br>• Type II: 26 renal units<br>• Type III: 8 renal units
Pelvicalyceal dilatation	SFU classification:<br>• SFU 1: 16 renal units<br>• SFU 2: 20 renal units<br>• SFU 3: 5 renal units<br>• SFU 4: 8 renal units<br>• SFU 5: 1 renal unit
Urinary drainage	Not reported
DRF	Data reported separately for:<br>• Non-surgically treated group (46/50 renal units): mean DRF: 48.8%<br>• Secondary surgically treated group (4/50 renal units): mean DRF: 36%
Follow-up duration, months	Mean 26, range 12-96

Loss of follow-up, n	Not reported
**Interventions**	
Planned interventions: 1) at the beginning (not clearly explained) US, ^{99m}Tc-MAG3 renal scintigraphy and presumably VCUG; 2) in the further course: repeated US (frequency not reported), ^{99m}Tc-MAG3 renal scintigraphy every 6-12 months	
VUR exclusion, procedure	All diseased renal units; VCUG
Functional imaging (type)	^{99m}Tc-MAG3 renal scintigraphy
Furosemide application for functional imaging	Not clearly declared
Bladder drainage during functional imaging	Yes
Initial threshold for primary surgical intervention	• Not precisely reported<br>• As indicated by the study:<br>- Sepsis<br>- Renal failure in solitary kidney
Co-interventions	Continuous antibiotic prophylaxis not routinely administered
Primary surgical intervention, n (%)	0 (0%)
Temporary diversions	Not reported
**Outcomes** (n=37 patients / n=50 renal units)	
Timing of last assessment	Variable
Ultrasound findings	• Resolution: 46 renal units<br>• Persistence: not reported<br>• Deterioration: not reported
Urinary drainage	Not reported
DRF	• General information: no deterioration in resolved cases<br>• Deterioration with DRF↓ ≥5% in at least 4 cases; not precisely reported; mean DRF in 4 renal units surgically treated: 34%
Threshold for secondary surgery	• Recurrent UTI under continuous antibiotic prophylaxis<br>• DRF↓ ≥5% during follow-up
Secondary surgical intervention, n (%)	4 renal units (8%)
Mode of surgery	Ureterocystoneostomy (tapered transtrigonal vesicoureteric reimplantation)
Timing of secondary surgery	After a mean follow-up of 58 months; range 32-80 months
UTI	Reported; data not separable
Other clinical complications	Not reported for study cohort; in the initial cohort 2 out of 54 patients: acute renal failure of single kidney
**Notes**	
Potential confounders not excluded; data on functional imaging not precisely reported; regarding selection of participants: some patients of the initial cohort not included	

Abbreviations: DRF, differential renal function; IVP, intravenous pyelogram; PM, primary megaureter; SFU, Society of Fetal Urology; ^{99m}Tc-MAG3, Technetium-99m-mercaptoacetyltriglycine; US, ultrasound; UTI, urinary tract infections; VCUG, voiding cystourethrogram; VUR, vesicoureteral reflux

Di Renzo (2013) [9]	
**Methods**	
Study design	Retrospective cohort study
Country	Italy
Setting	Monocentric, tertiary center
Power analysis	Not reported
Funding sources	Not reported
Conflict of interest	Not reported
Study dates	1990-2005
**Participants – INCLUSION and EXCLUSION CRITERIA, COMORBIDITIES, POTENTIAL CONFOUNDERS**	
Inclusion criteria	Diagnosis of primary megaureter with distal ureteral dilatation ≥7mm
Exclusion criteria	VUR, urinary tract duplication, ectopic ureter, bladder or urethral pathology, bladder outlet obstruction or ureterocele
Comorbidities	Not reported
Confounders	In the initial cohort (including patients with primary surgery 1 patient with solitary kidney)<br>Bilateral cases
**Definitions**	
Megaureter in terms of ureteral dilatation (mm)	Distal ureteral dilatation ≥ 7mm
Megaureter in terms of IVP	Grading due to Pfister Hendren classification [2]
Pelvicalyceal dilatation	Grading due to SFU classification [8]; grades I-IV
Urinary drainage (functional imaging)	Clearance half time interpretation
DRF (functional imaging)	DRF <40%: impaired
Obstruction	Clearance half time interpretation >20 min
Resolution	Residual pelvicalyceal dilatation <SFU II + retrovesical ureteral dilatation <7 mm
**Participants – GROUP CHARACTERISTICS**	
Total number of participants, n	75
Number of renal units, n (total / left / right / bilateral)	88 / 43 / 19 / 13
Female, n (%)	12 (16%)
Age at diagnosis of PM	42 patients prenatally<br>14 patients during neonatal period<br>19 patients beyond neonatal period
Mean / median age of study participants (range)	Not reported for the whole cohort
Ureteral dilatation, mm	Not reported
Ureteral dilatation, assessment by IVP	Pfister-Hendren classification:<br>• Type I or II: 68 renal unis<br>• Type III: 6 renal units
Pelvicalyceal dilatation	SFU classification:<br>• SFU I or II: 39 renal units<br>• SFU ≥III: 35 renal units
Urinary drainage	Data reported only for n=64 renal units, because renal scintigraphy not performed in mild pelvicalyceal dilatation<br>• Nonobstructive: 55 renal units<br>• Intermediate/obstructive: 9 renal units
DRF	Data reported only for n=64 renal units, because renal scintigraphy not performed in mild pelvicalyceal dilatation<br>• <40%: 3 renal units<br>• ≥40%: 61 renal units
Follow-up duration, months	Median 120, range 60-180
Loss of follow-up, n	Not reported

Interventions	
Planned interventions: 1) initial assessment: US, VCUG and ^{99m}Tc-MAG3 or ^{99m}Tc-DMSA renal scintigraphy; 2) follow-up: ultrasound and renal scintigraphy individually and according to physician's discretion	
VUR exclusion, procedure	All diseased renal units; VCUG or voiding urosonography
Functional imaging (type)	^{99m}Tc-DMSA or ^{99m}Tc-MAG3 renal scintigraphy
Furosemide application for functional imaging	Not reported
Bladder drainage during functional imaging	Not reported
Initial threshold for primary surgical intervention	• Not precisely reported<br>• As indicated by the study:<br>- Urosepsis<br>- Renal failure in solitary kidney<br>- Other symptoms, not specified<br>- Severe urinary tract dilatation + DRF↓ + obstructive urinary drainage pattern<br>- Severe bilateral pelvicalyceal dilatation<br>- Severe pelvicalyceal dilatation in solitary kidney
Co-interventions	Continuous antibiotic prophylaxis individually / case by case
Primary surgical intervention, n (%)	14/88 renal units (16%)
Temporary diversions	2 patients: temporary urinary diversion (primary surgery group)
**Outcomes** (n=63 patients / n=74 renal units; 12 patients / 14 renal units due to primary surgery excluded; furthermore, outcome parameters with missing data for some patients / renal units)	
Timing of last assessment	Variable
US findings	• Resolution: 47 renal units<br>• Persistence / Improvement not separable: mild hydroureteronephrosis: 7 renal units<br>• Deterioration: not reported
Urinary drainage	Not reported
DRF	• >40%: 54 renal units (non-surgically managed) + at least 9 renal units (secondary surgery, preoperatively assessed)<br>• Deterioration: 4 renal units
Threshold for secondary surgery	• Pelvicalyceal dilatation SFU IV + obstructive urinary drainage pattern or persistent symptoms or DRF↓ ≥5% during follow-up
Secondary surgical intervention, n (%)	20/74 renal units (27%)
Mode of surgery	Not reported
Timing of secondary surgery	8-96 months after diagnosis
UTI	Reported, data not separable
Other clinical complications	Flank pain reported, data not separable
Notes	
Potential confounders not excluded; discrepancies between data in tables and text, several data not clearly separable	

Abbreviations: DRF, differential renal function; IVP, intravenous pyelogram; PM, primary megaureter; SFU, Society of Fetal Urology; ^{99m}Tc-DMSA, Technetium-99m-dimercaptosuccinyl-acid; ^{99m}Tc-MAG3, Technetium-99m-mercaptoacetyltriglycine; US, ultrasound; UTI, urinary tract infections; VCUG, voiding cystourethrogram; VUR, vesicoureteral reflux

**Gimpel (2010)** [10]	
**Methods**	
Study design	Retrospective cohort study
Country	Germany
Setting	Monocentric, tertiary center
Power analysis	Not reported
Funding sources	Not reported
Conflict of interest	Not reported
Study dates	1994-2006
**Participants – INCLUSION and EXCLUSION CRITERIA, COMORBIDITIES, POTENTIAL CONFOUNDERS**	
Inclusion criteria	Diagnosis of primary megaureter, follow-up of at least 12 months, at least 1 US and 1 renal isotope scan
Exclusion criteria	Secondary megaureters; megaureters with type A renogram curve (i. e. non-obstructive megaureters); presence of duplicated kidneys or duplicated ureters
Comorbidities	2 patients: ipsilateral pelvi-ureteric junction obstruction<br>4 patients: ipsilateral renal hypo-/dysplasia<br>2 patients: contralateral pelvi-ureteric junction obstruction<br>2 patients: contralateral VUR<br>1 patient: contralateral renal agenesis<br>5 patients: extrarenal anomalies
Confounders	2 patients: ipsilateral pelvi-ureteric junction obstruction<br>4 patients: ipsilateral renal hypo-/dysplasia<br>2 patients: contralateral pelvi-ureteric junction obstruction<br>2 patients: contralateral VUR<br>1 patient: contralateral renal agenesis<br>Bilateral cases<br>In some patients VUR not excluded
**Definitions**	
Megaureter in terms of ureteral dilatation (mm)	Congenital ureteral dilatation
Megaureter in terms of IVP	Not applicable, IVP not performed
Pelvicalyceal dilatation	Grading due Hofmann classification [11]; grades I-IV
Urinary drainage (functional imaging)	Grading based on O`Reilly criteria [3, 4]:<br>• Type A: normal<br>• Type B: obstructive<br>• Type C: dilated non-obstructive<br>• Type D: partially obstructive
DRF (functional imaging)	DRF <45%: impaired renal function
Obstruction	O`Reilly type B
Resolution	O`Reilly type A on follow-up<br>or regression of ureteral + pelvicalyceal dilatation on US, if no serial renogram
**Participants – GROUP CHARACTERISTICS**	
Total number of participants, n	49
Number of renal units, n (total / left / right / bilateral)	56 / 28 / 14 / 7
Female, n (%)	14 (29%)
Age at diagnosis of PM	29 patients prenatally<br>18 patients incidentally, of whose: mean age 10±19 months, range 1 week to 77 months<br>2 patients: not reported
Mean / median age of study participants (range)	Not reported
Ureteral dilatation, mm	Whole cohort (including patients with primary surgery):<br>Mean 11.5±5 mm, range 4-24 mm
Ureteral dilatation, assessment by IVP	Not applicable, IVP not performed
Pelvicalyceal dilatation	Hofmann classification (whole cohort (including patients with primary surgery):<br>• Grade 0: 1 renal unit<br>• Grade I: 10 renal units

	• Grade II: 39 renal units<br>• Grade III: 5 renal units<br>• Grade IV: 1 renal unit
Urinary drainage	Whole cohort (including patients with primary surgery):<br>• Type A: 2 renal units<br>• Type B: 6 renal units<br>• Type C: 38 renal units<br>• Type D: 6 renal units
DRF	Whole cohort (including patients with primary surgery):<br>• DRF >45%: not separable<br>• DRF <45%: 7 renal units
Follow-up duration, months	Mean 47±30, range 12-78
Loss of follow-up, n	In the initial cohort some patients with loss of follow-up or incomplete data
**Interventions**	
Planned interventions: No detailed protocol reported; mean number of US 6.4±3.3; mean number of renal scintigraphy: 2.9±2.2; indications for initial ^{99m}Tc-MAG3 renal scintigraphy: intrarenal pelvis >15 mm or intrarenal pelvis 12-15 mm with grade II pelvicalyceal dilatation; indications for scintigraphy in the further course: initial drainage pattern type B, D or C without improvement on US	
VUR exclusion, procedure	41/49 patients; boys: VCUG, girls: voiding urosonography
Functional imaging (type)	^{99m}Tc-MAG3 renal scintigraphy, in some cases magnetic resonance urography
Furosemide application for functional imaging	Yes; furosemide 20 min after ^{99m}Tc-MAG3 injection; 1 mg/kg for infants, 0.5 mg/kg for older children, maximum 40 mg
Bladder drainage during functional imaging	Not routinely
Initial threshold for primary surgical intervention	• Not precisely reported<br>• As indicated by the study:<br>- Initial obstructive or equivocal urinary drainage pattern
Co-interventions	Continuous antibiotic prophylaxis in 30/49 patients
Primary surgical intervention, n (%)	4/56 renal units (7%)
Temporary diversions	2 patients: percutaneous nephrostomy<br>1 patient: temporary external urine diversion and splinting after ureterocystoneostomy with early stenosis
**Outcomes** (n=45 patients / n=52 renal units; 4 patients / 4 renal units due to primary surgery excluded; furthermore, outcome parameters with missing data for some patients / renal units)	
Timing of last assessment	Variable
US findings	• Resolution: 36 renal units<br>• Persistence: not precisely reported<br>• Improvement: not precisely reported<br>• Deterioration: not precisely reported
Urinary drainage	(15 renal units not examined):<br>• Resolution: 2 renal units<br>• Persistence: 20 renal units<br>• Improvement: 7 renal units<br>• Deterioration: 6 renal units
DRF	Data not precisely separable
Threshold for secondary surgery	• Proven relevant obstruction (renogram)<br>• Major complications
Secondary surgical intervention, n (%)	9/52 renal units (17%)
Mode of surgery	Ureterocystoneostomy (different techniques) with or without preceding temporary diversion
Timing of secondary surgery	At age of 36±22 months, range 10.7-72.3
UTI	33/49 patients (17/49 >1 event)
Other clinical complications	1 patient: arterial hypertension; 1 patient: abdominal pain; 0 patients: chronically impaired glomerular renal function
Notes	
Potential confounders not excluded; some outcome data not separable	

Abbreviations: DRF, differential renal function; IVP, intravenous pyelogram; PM, primary megaureter; ^{99m}Tc-MAG3, Technetium-99m-mercaptoacetyltriglycine; US, ultrasound; UTI, urinary tract infections; VCUG, voiding cystourethrogram; VUR, vesicoureteral reflux

Liu (1994) [12]	
**Methods**	
Study design	Not clearly reported, presumably retrospective
Country	United Kingdom
Setting	Monocentric, tertiary center
Power analysis	Not reported
Funding sources	Not reported
Conflict of interest	Not reported
Study dates	1988-1991
**Participants – INCLUSION and EXCLUSION CRITERIA, COMORBIDITIES, POTENTIAL CONFOUNDERS**	
Inclusion criteria	Primary non-refluxing megaureter, prenatal diagnosis with postnatal confirmation
Exclusion criteria	VUR, secondary megaureter
Comorbidities	Not precisely reported; at least one patient with solitary kidney<br>Not reported
Confounders	At least one patient with solitary kidney<br>Bilateral cases
**Definitions**	
Megaureter in terms of ureteral dilatation (mm)	Wide ureter
Megaureter in terms of IVP	Not applicable, IVP not performed
Pelvicalyceal dilatation	Not reported
Urinary drainage (functional imaging)	Interpretation based on T_{75} (time to clear up 75% of activity):<br>• T_{75} <5 min: normal<br>• T_{75} 5-10 min and <50% of activity retained in upper urinary tract after 40 min: mild to moderate drainage delay<br>• T_{75} 5-10 min and <50% of activity retained in upper urinary tract after 40 min: poor or no drainage
DRF (functional imaging)	DRF <40%: impaired
Obstruction	Not reported
Resolution	Not precisely defined, resolution of ureteral dilatation on US
**Participants – GROUP CHARACTERISTICS**	
Total number of participants, n	53
Number of renal units, n (total / left / right / bilateral)	67 / 29 / 10 / 14
Female, n (%)	18 (34%)
Age at diagnosis of PM	Prenatal diagnosis in all patients
Mean / median age of study participants (range)	Not reported
Ureteral dilatation, mm	Whole cohort (including patients with primary surgery):<br>Mean 8.4±3.5 mm<br>• <6 mm: 18 renal units<br>• 6-10 mm: 35 renal units<br>• >10 mm: 14 renal units
Ureteral dilatation, assessment by IVP	Not applicable, IVP not performed
Pelvicalyceal dilatation	Not reported
Urinary drainage	Whole cohort (including patients with primary surgery):<br>• T_{75} <5 min: 27 renal units<br>• T_{75} <5-10 min, retention <50%: 25 renal units<br>• T_{75} >10 min, retention >50%: 50 renal units
DRF	Whole cohort (including patients with primary surgery):<br>• DRF >40%: at least 52 renal units<br>• DRF 10%: 1 renal unit<br>• DRF 10-40%: unknown, data not reported for all renal units
Follow-up duration, months	Mean 37, range 14-60

Loss of follow-up, n	Not reported
**Interventions**	
Planned interventions: 1) at the beginning: US, VCUG; ^{99m}Tc-DTPA renal scintigraphy; US in newborn period, at the age of 3 months and 12 months, thereafter yearly; in case of "massive" ureteral or pelvicalyceal dilatation also at 6 months; 2) follow-up: ^{99m}Tc-DTPA renal scintigraphy at 3 months, 6 months, 12 months, 24 months; in case of symptoms variations; in case of resolution of hydroureteronephrosis only US	
VUR exclusion, procedure	All diseased renal units; VCUG
Functional imaging (type)	^{99m}Tc-DTPA renal scintigraphy
Furosemide application for functional imaging	Yes; 0.2 mg/kg at 20 min
Bladder drainage during functional imaging	Yes
Initial threshold for primary surgical intervention	• Not reported<br>• As indicated by the study<br>- Markedly impaired DRF
Co-interventions	Continuous antibiotic prophylaxis in all patients
Primary surgical intervention, n (%)	1 (2%)
Temporary diversions	Not reported
**Outcomes** (n=52 patients / n=66 renal units; 1 patient / 1 renal unit due to primary surgery excluded)	
Timing of last assessment	Variable
Ultrasound findings	Data reported only for 56 non-surgically managed patients:<br>• Resolution: 23 renal units<br>• Persistence: 22 renal units<br>• Improvement: 6 renal units<br>• Deterioration: 5 renal units
Urinary drainage	Data not separable
DRF	Data not reported for all patients:<br>• DRF stable: in 23 resolved renal units<br>• Deterioration: 7 renal units
Threshold for secondary surgery	• Symptomatic UTI under continuous antibiotic prophylaxis<br>• DRF↓ to <40% or >10% during follow-up
Secondary surgical intervention, n (%)	10 renal units (15%)
Mode of surgery	Ureterocystoneostomy (plication and reimplantation)
Timing of secondary surgery	For whole cohort (including 1 patient with primary surgery): at the age of 3-15 months; mean age for infection group: 9 months; mean age for DRF↓ group: 6 months
UTI	3 patients
Other clinical complications	Not reported
**Notes**	
Potential confounders not excluded, several outcome data not separable	

Abbreviations: DRF, differential renal function; IVP, intravenous pyelogram; PM, primary megaureter; ^{99m}Tc-DTPA, Technetium-99m-diethylenetriamine-pentaacetic-acid; US, ultrasound; UTI, urinary tract infections; VCUG, voiding cystourethrography; VUR, vesicoureteral reflux

Oliveira (2000) [13]	
**Methods**	
Study design	Prospective observational study
Country	Brazil
Setting	Monocentric, tertiary center
Power analysis	Not reported
Funding sources	Partially supported by Fundação de Amparo a Pesquisa do Estado de Minas Gerais (FAPEMIG), Conselho de Desenvolvimento Científico e Tecnológico (CNPq) and Pró-Reitoria de Pesquisa-UFMG
Conflict of interest	Not reported
Study dates	01/1985-07/1995
**Participants – INCLUSION and EXCLUSION CRITERIA, COMORBIDITIES, POTENTIAL CONFOUNDERS**	
Inclusion criteria	Prenatally diagnosed pelvicalyceal dilatation, postnatal diagnosis of primary non-refluxing megaureter
Exclusion criteria	VUR, urethral valves, severe bilateral pelvicalyceal dilatation
Comorbidities	1 patient: atrial septal defect and congenital scoliosis
Confounders	Bilateral cases
**Definitions**	
Megaureter in terms of ureteral dilatation (mm)	Not reported
Megaureter in terms of IVP	IVP performed, grading not reported
Pelvicalyceal dilatation	Not reported
Urinary drainage (functional imaging)	Not reported
DRF (functional imaging)	Not reported
Obstruction	Not reported
Resolution	Not precisely defined, regression of US findings / dilatation
**Participants – GROUP CHARACTERISTICS**	
Total number of participants, n	8
Number of renal units, n (total / left / right / bilateral)	11 / 3 / 2 / 3
Female, n (%)	3 (38%)
Age at diagnosis of PM	Prenatal diagnosis, postnatal confirmation of diagnosis during neonatal period
Mean / median age of study participants (range)	Not reported
Ureteral dilatation, mm	(2 renal units initially not examined, therefore n=9)<br>Mean 13.6 mm, range 7.5-21 mm
Ureteral dilatation, assessment by IVP	Not reported
Pelvicalyceal dilatation	Present in all renal units, grading not reported
Urinary drainage	Not reported
DRF	DRF >40%: 11 renal units
Follow-up duration, months	Median 75.5, mean 53, range 37-124
Loss of follow-up, n	Not reported
**Interventions**	
Planned interventions: 1) at the beginning: US and VCUG; ^{99m}Tc-DMSA, ^{99m}Tc-DTPA renal scintigraphy and/or IVP; 2) in the further course: annually US and ^{99m}Tc-DTPA renal scintigraphy until improvement of ureteral dilatation; in case of symptoms promptly performance of US and ^{99m}Tc-DTPA renal scintigraphy	
VUR exclusion, procedure	All diseased renal units; VCUG
Functional imaging (type)	^{99m}Tc-DMSA or ^{99m}Tc-DTPA renal scintigraphy
Furosemide application for functional imaging	Not clearly declared
Bladder drainage during functional imaging	Not reported
Initial threshold for primary surgical intervention	Not reported
Co-interventions	Continuous antibiotic prophylaxis in all patients

Primary surgical intervention, n (%)	0 (0%)
Temporary diversions	Not applicable, no surgical intervention
**Outcomes** (n=8 patients / n=11 renal units)	
Timing of last assessment	Variable
Ultrasound findings	(2 renal units initially not examined, therefore n=9)<br>Reported for ureteral dilatation:<br>• Resolution: 7 renal units<br>• Persistence: 0 renal units<br>• Improvement: 0 renal units<br>• Deterioration: 2 renal units
Urinary drainage	• Resolution: not precisely reported<br>• Persistence: not precisely reported<br>• Improvement: 11 renal units<br>• Deterioration: 0 renal units
DRF	• DRF > 40%: 11 renal units<br>• Deterioration: 0 renal units
Threshold for secondary surgery	Not reported
Secondary surgical intervention, n (%)	0 (0%)
Mode of surgery	Not applicable
Timing of secondary surgery	Not applicable
UTI	Not reported
Other clinical complications	2 patients: arterial hypertension; 0 patients: chronically impaired glomerular renal function
**Notes**	
Potential confounders not excluded, several outcome data not reported in a quantitative manner	

Abbreviations: DRF, differential renal function; IVP, intravenous pyelogram; PM, primary megaureter; ^{99m}Tc-DMSA, Technetium-99m-dimercaptosuccinyl-acid; ^{99m}Tc-DTPA, Technetium-99m-diethylenetriamine-pentaacetic-acid, US, ultrasound; UTI, urinary tract infections; VCUG, voiding cystourethrogram; VUR, vesicoureteral reflux

**Stehr (2000)** [14]	
**Methods**	
Study design	Not clearly reported, presumably retrospective
Country	Germany
Setting	Monocentric, tertiary center
Power analysis	Not reported
Funding sources	Not reported
Conflict of interest	Not reported
Study dates	1996-1999
**Participants – INCLUSION and EXCLUSION CRITERIA, COMORBIDITIES, POTENTIAL CONFOUNDERS**	
Inclusion criteria	Primary non-refluxing megaureter
Exclusion criteria	VUR, ureteroceles, ectopic ureter, subvesical outlet obstruction “or associated symptoms” (not specified)
Comorbidities	4 patients: contralateral VUR<br>Extrarenal comorbidities not reported
Confounders	4 patients: contralateral VUR<br>Bilateral cases
**Definitions**	
Megaureter in terms of ureteral dilatation (mm)	Not reported
Megaureter in terms of IVP	Grading due to Pfister Hendren classification [2]
Pelvicalyceal dilatation	Grading due to SFU classification [8]; grades 0-4
Urinary drainage (functional imaging)	Interpretation of urinary drainage:<br>• >50% 30 min after ^{99m}Tc-MAG3 injection: non-obstructive<br>• >60% 20 min after furosemide injection: functionally obstructive<br>• 40-60% 20 min after furosemide injection: equivocal<br>• <40% 20 min after furosemide injection: obstructive
DRF (functional imaging)	DRF <40%: impaired
Obstruction	Urinary drainage <40% 20 min after furosemide injection in context with ^{99m}Tc-MAG3 renal scintigraphy
Resolution	Not reported
**Participants – GROUP CHARACTERISTICS**	
Total number of participants, n	42
Number of renal units, n (total / left / right / bilateral)	53 / 27 / 10 / 8
Female, n (%)	5 (14%)
Age at diagnosis of PM	15 patients prenatally<br>21 patients during neonatal period<br>6 patients postnatally at age of 3-8 years
Mean / median age of study participants (range)	Not reported
Ureteral dilatation, mm	Whole cohort (including 1 patient with primary surgery)<br>Range 5 - 14 mm
Ureteral dilatation, assessment by IVP	Whole cohort (including 1 patient with primary surgery)<br>Pfister-Hendren classification:<br>• Type IA or IB: 14 renal units<br>• Type II: 24 renal units<br>• Type III: 15 renal units
Pelvicalyceal dilatation	Whole cohort (including 1 patient with primary surgery)<br>SFU classification:<br>• SFU 0: 2 renal units<br>• SFU 1: 7 renal units<br>• SFU 2: 19 renal units<br>• SFU 3: 23 renal units<br>• SFU 4: 2 renal units
Urinary drainage	• Functional obstruction: 9 renal units<br>• Equivocal drainage: 34 renal units<br>• Obstructive: 9 renal units

DRF	Whole cohort (including 1 patient with primary surgery) (not reported if mean or median)<br>• Functional obstruction (n=9): DRF 56.3%, range 47-80%<br>• Equivocal drainage (n=34): 47%, range 26-74%<br>• Obstruction (n=19): 43.6%, range 20-60%
Follow-up duration, months	Mean 22.1, range 5-48
Loss of follow-up, n	Not reported
**Interventions**	
Planned interventions: 1) initial postnatal assessment: US (in severe cases at the beginning monthly); VCUG; IVP; ^{99m}Tc-MAG3 renal scintigraphy; 2) follow-up: serial sonography in all patients; serial ^{99m}Tc-MAG3 renal scintigraphy in 36 patients; no serial IVP	
VUR exclusion, procedure	All diseased renal units; VCUG
Functional imaging (type)	^{99m}Tc-MAG3 renal scintigraphy
Furosemide application for functional imaging	Yes; 1 mg/kg, maximum 20 mg, if urinary drainage <50% 30 min after ^{99m}Tc-MAG3 injection
Bladder drainage during functional imaging	Not reported
Initial threshold for primary surgical intervention	Initial obstructive urinary drainage and DRF <40%
Co-interventions	Continuous antibiotic prophylaxis not administered in all patients, but in all neonates
Primary surgical intervention, n (%)	1 (3%)
Temporary diversions	Occasionally ureterostomy in surgically treated patients
**Outcomes** (n=41 patients / n=52 renal units; 1 patient / 1 renal unit due to primary surgery excluded)	
Timing of last assessment	Variable
Ultrasound findings	Data regarding pelvicalyceal dilatation, data not clearly separable (data reported for all patients including surgically managed patients):<br>• Decrease/improvement in all patients<br>• SFU grade 0: 4 renal units<br>• SFU grade 1: 15 renal units<br>• SFU grade 2: 28 renal units<br>• SFU grade 3: 5 renal units<br>• SFU grade 4: 1 renal unit
Urinary drainage	• No obstruction: 5 renal units<br>• Remaining data not separable
DRF	• DRF stable in all renal units<br>• Deterioration: 0 renal units
Threshold for secondary surgery	• Normal DRF and at least equivocal obstructive urinary drainage with no improvement or deterioration of urinary drainage and/or DRF↓ during follow-up<br>• Any symptoms (e. g. UTI, calculus)
Secondary surgical intervention, n (%)	4 renal units (8%)
Mode of surgery	Ureterocystoneostomy with or without previous ureterostomy
Timing of secondary surgery	At the age of 6, 10, 36 and 60 months
UTI	13 patients
Other clinical complications	1 patient: urolithiasis; 2 patients: abdominal pain
**Notes**	
Potential confounders not excluded; several outcome data not separable	

Abbreviations: DRF, differential renal function; IVP, intravenous pyelogram; SFU, Society of Fetal Urology; ^{99m}Tc-MAG3, Technetium-99m-mercaptoacetyltriglycine; US, ultrasound; UTI, urinary tract infections; VCUG, voiding cystourethrogram; VUR, vesicoureteral reflux

Appendix 5: Definitions and classifications of non-refluxing primary megaureter in the included studies

Included studies	Definitions and classifications								
	Primary non-refluxing megaureter				Pelvicalyceal dilatation	Functional imaging (^{99m}Tc-DMSA / ^{99m}Tc-DTPA / ^{99m}Tc-MAG3 renal scintigraphy)			Resolution
	Secondary megaureter excluded	VUR excluded	Predefined ureteral dilatation	Megaureter (IVP)	Classification	Urinary drainage	DRF	Obstruction	
**Antón-Pacheco Sanchez (1995)**	Yes	Yes	NR	Pfister-Hendren	Pfister-Hendren	O`Reilly renogram:<br>Types I / II / IIIa / III b<br>Clearance half time:<br><15 min: non-obstructive<br>15-20 min: partially obstructive<br>>20 min: obstructive	<30%: poor<br>30-40%: moderate<br>>40%: good	O`Reilly type II	(Not precisely defined)<br><br>Normalization of US findings
**Arena (1998)**	Yes	Yes	NR	Beurton	Beurton	Diuretic test:<br>Positive: non-obstructive<br>Equivocal<br>Negative: obstructive<br>= tracer accumulation >50% 20 min after furosemide application	≤40%: impaired	Negative diuretic test	(Not precisely defined)<br><br>Regression in dilatation
**Calisti (2008)**	Yes	Yes	Distal ureteral dilatation ≥10 mm	Pfister-Hendren	SFU 1-5	NR	NR; due to pooling only early phase of ^{99m}Tc-MAG3 parenchymal uptake considered	NR	(Not precisely defined)<br><br>Resolution of dilatation
**Di Renzo (2013)**	Yes	Yes	Distal ureteral dilatation ≥7 mm	Pfister-Hendren	SFU I-IV	Clearance half time >20 min: obstructive	<40%: impaired	Clearance half time >20 min	Residual pelvicalyceal dilatation <SFU II + retrovesical ureteral dilatation <7 mm
**Gimpel (2010)**	Yes	Yes*	Congenital ureteral dilatation**	NA<br>(no IVP)	Hofmann I-IV	O`Reilly renogram:<br>Types A / B / C / D	<45%: impaired	O`Reilly type B	O`Reilly type A on follow-up or regression of ureteral + pelvicalyceal dilatation on US, if no serial renogram

Included studies	Definitions and classifications								
	Primary non-refluxing megaureter				Pelvicalyceal dilatation	Functional imaging (^{99m}Tc-DMSA / ^{99m}Tc-DTPA / ^{99m}Tc-MAG3 renal scintigraphy)			Resolution
	**Secondary megaureter excluded**	**VUR excluded**	**Predefined ureteral dilatation**	**Megaureter (IVP)**	**Classification**	**Urinary drainage**	**DRF**	**Obstruction**	
**Liu (1994)**	Yes	Yes	Wide ureter***	NA<br>(no IVP performed)	NR	T_{75} (time to clear up 75% of activity) <5 min: normal<br>T_{75} 5-10 min and <50% of activity retained in upper tract after 40 min: mild to moderate drainage delay<br>T_{75} >10 min and >50% of activity retained in upper tract after 40 min: poor or no drainage	<40%: impaired	NR	(Not precisely defined)<br><br>Resolution of ureteral dilatation on US
**Oliveira (2000)**	Yes	Yes	NR	NR<br>(IVP performed)	NR	NR	NR	NR	(Not precisely defined)<br><br>Regression of US findings / dilatation
**Stehr (2002)**	Yes	Yes	NR****	Pfister-Hendren	SFU 0-4	Urinary drainage:<br>>50% 30 min after MAG3-injection: non-obstructive<br>Urinary drainage 20 min after furosemide injection:<br>>60%: functionally obstructive<br>40-60%: equivocal<br><40%: obstructive	<40%: impaired	Urinary drainage <40% 20 min after furosemi-de injection	NR

Abbreviations: DRF, differential renal function; NA, not applicable; NR, not reported; IVP, intravenous pyelogram; US, ultrasound; SFU, Society for Fetal Urology; ^{99m}Tc-DMSA, Technetium-99m-dimercaptosuccinyl-acid; ^{99m}Tc-DTPA, Technetium-99m-diethylenetriamine-pentaacetic-acid; ^{99m}Tc-MAG3, Technetium-99m-mercaptoacetyltriglycine; VUR, vesicoureteral reflux

*In 8 out of 49 patients VUR not excluded. **Lowest ureteral diameter in the study sample assigned as megaureter: 4 mm. ***Lowest ureteral diameter in the study sample assigned as megaureter: 6 mm. ****Lowest ureteral diameter in the study sample assigned as megaureter: 5 mm.

Used classification systems regarding:

1) IVP: Pfister-Hendren [2] and Beurton [6] classification
2) Pelvicalyceal dilatation: SFU [8] and Hofmann [11] classification
3) Drainage pattern / renogram: O`Reilly [3, 4]

Appendix 6: Characteristics of non-refluxing primary megaureter at enrolment in the included studies

**Included studies**				**Diagnostic findings at enrolment** (reported for renal units)			
	**Study cohort**			**Sonographic finding**		**Renal scintigraphy**<br>(^{99m}Tc-DMSA / ^{99m}Tc-DTPA / ^{99m}Tc-MAG3 scintigraphy)	
	**Renal units included in study, n**	**Renal units undergoing primary surgery, n**	**Renal units eligible for systematic review, n**	**Ureteral dilatation, n**	**Pelvicalyceal dilatation, n**	**Differential renal function, n**	**Urinary drainage, n**
**Antón-Pacheco Sanchez (1995)**	26<br><br>(23 patients)	0	**26**<br><br>(23 patients)	NR in mm<br><br>Pfister-Hendren (IVP):<br>Moderate: 22<br>Ureteral dilatation not precisely reported: 4	Present in 26; grading not precisely reported for all renal units	30-40%: 2<br>>40%: 24	Curves and t½:<br>IIIa, t½ <15 min: 13<br>IIIa, t½ 15-20 min: 5<br>IIIa, t½ <15 min: 1<br>IIIb, t½ <15 min: 6<br>IIIb, t½ <15 min: 1
**Arena (1998)**	24<br><br>(22 patients)	1<br><br>(1 patient)	**23**<br><br>(21 patients)	Not reported in mm<br><br>Beurton (IVP):<br>Type I: 2<br>Type II: 9<br>Type III: 12	Not precisely reported<br>for all renal units<br><br>See also Beurton	>40%: 23	Non-obstructive: 19<br>Equivocal: 2<br>Obstructive: 2
**Calisti (2008)**	50<br><br>(37 patients)	0	**50**<br><br>(37 patients)	Data reported separately for:<br><br>1) only non-surgically treated group (46/50): mean ureteral diameter 10.9 mm<br><br>2) secondary surgically treated group (4/50): mean ureteral diameter 14.7 mm<br><br>Pfister-Hendren (IVP):<br>Type I: 16<br>Type II: 26<br>Type III: 8	SFU 1: 16<br>SFU 2: 20<br>SFU 3: 5<br>SFU 4: 8<br>SFU 5: 1	Data reported separately for:<br><br>1) non-surgically treated group (46/50):<br>mean DRF 48.8%<br><br>ureteral diameter 10.9 mm<br><br>2) secondary surgically treated group (4/50):<br>mean DRF 36%	NR

**Included studies**	**Diagnostic findings at enrolment** (reported for renal units)						
	**Study cohort**			**Sonographic finding**		**Renal scintigraphy**<br>(^{99m}Tc-DMSA / ^{99m}Tc-DTPA / ^{99m}Tc-MAG3 scintigraphy)	
	**Renal units included in study, n**	**Renal units undergoing primary surgery, n**	**Renal units eligible for systematic review, n**	**Ureteral dilatation, n**	**Pelvicalyceal dilatation, n**	**Differential renal function, n**	**Urinary drainage, n**
**Di Renzo (2013)**	88<br><br>(75 patients)	14<br><br>(12 patients)	**74**<br><br>(63 patients)	NR in mm<br><br>Pfister-Hendren (IVP):<br>Type I + II: 68<br>Type III: 6	SFU I or II: 39<br>SFU ≥III: 35	(n=64, in patients with mild pelvicalyceal dilatation in some cases renal scan not performed)<br><br><40%: 3<br>≥40%: 61	(n=64, in patients with mild pelvicalyceal dilatation in some cases renal scan not performed)<br><br>Nonobstructive: 55<br>Intermediate/obstructive: 9
**Gimpel (2010)***	56<br><br>(49 patients)	4<br><br>(4 patients)	**52**<br><br>(45 patients)	Data of non-surgically treated not separable from renal units undergoing primary surgery<br><br>Whole cohort:<br>Mean 11.5±5 mm<br>(range 4-24 mm)	Data of non-surgically treated not separable from renal units undergoing primary surgery<br><br>Whole cohort:<br>Hofmann:<br>Grade 0: 1<br>Grade I: 10<br>Grade II: 39<br>Grade III: 5<br>Grade IV: 1	Data of non-surgically treated not separable from renal units undergoing primary surgery<br><br>Whole cohort:<br><45%: 7<br>>45%: not separable	Normal: 2<br>Obstructive: 6<br>Dilated non-obstructive: 38<br>Partially obstructive: 6
**Liu (1994)**	67<br><br>(53 patients)	1<br><br>(1 patient)	**66**<br><br>(52 patients)	Data of non-surgically treated not separable from renal units undergoing primary surgery<br><br>Whole cohort:<br>Mean 8.4±3.5 mm<br><6 mm: 18<br>6-10 mm: 35<br>>10 mm: 14	NR	>40%: at least 52<br>10%: 1<br>10-40%: unknown; data not reported for all renal units<br><br>6 further renal units treated by secondary surgery:<br>mean initial DRF 45.3%, range 39-56%	Data of non-surgically treated not separable from renal units undergoing primary surgery<br><br>Whole cohort:<br><br>1) Non-obstructive drainage pattern (T_{75} <5 min): 27<br><br>2) Mild to moderate drainage delay (T_{75} <5-10 min, retention <50%): 25

Included studies	Diagnostic findings at enrolment (reported for renal units)						
	Study cohort			Sonographic finding		Renal scintigraphy (^{99m}Tc-DMSA / ^{99m}Tc-DTPA / ^{99m}Tc-MAG3 scintigraphy)	
	**Renal units included in study, n**	**Renal units undergoing primary surgery, n**	**Renal units eligible for systematic review, n**	**Ureteral dilatation, n**	**Pelvicalyceal dilatation, n**	**Differential renal function, n**	**Urinary drainage, n**
							3) Poor or no drainage (T_{75} >10 min, retention >50%): 15
**Oliveira (2000)**	11<br>(8 patients)	0	**11**<br>(8 patients)	(n=9; 2 renal units [1 patient] not examined initially)<br>Mean 13.6 mm, range 7.5-21 mm	Present in all renal units; grading NR	>40%: 11	NR
**Stehr (2002)**	53<br>(42 patients)	1<br>(1 patient)	**52**<br>(41 patients)	Data of non-surgically treated not separable from renal units undergoing primary surgery<br>Whole cohort:<br>Range: 5-14 mm<br>Pfister-Hendren (IVP):<br>Grade IA or IB: 14<br>Grade II: 24<br>Grade III: 15	Data of non-surgically treated not separable from renal units undergoing primary surgery<br>Whole cohort:<br>SFU 0: 2<br>SFU 1: 7<br>SFU 2: 19<br>SFU 3: 23<br>SFU 4: 2	Data of non-surgically treated not separable from renal units undergoing primary surgery (not reported if mean or median)<br>1) functional obstruction, 9: 56.3%, range 47-80%<br>2) equivocal drainage, 34: 47%, range: 26-74%<br>3) obstruction, 10: 43.6%, range 20-60%	1) functional obstruction: 9<br>2) equivocal drainage: 34<br>3) obstructive: 9

*Additional, non-published data gathered by personal contact.

Abbreviations: DRF, differential renal function; NR, not reported; IVP, intravenous pyelogram; SFU, Society for Fetal Urology; ^{99m}Tc-DMSA, Technetium-99m-dimercaptosuccinyl-acid; ^{99m}Tc-DTPA, Technetium-99m-diethylenetriamine-pentaacetic-acid; ^{99m}Tc-MAG3, Technetium-99m-mercaptoacetyltriglycine

Appendix 7: Indications for surgical intervention

Included studies	Indications	
	**Primary surgery**	**Secondary surgery**
**Antón-Pacheco Sanchez (1995)**	NR	(Predefined criteria NR)<br>• Abdominal pain + change to obstructive urinary drainage pattern
**Arena (1998)**	(Predefined criteria NR)<br>• Severe ureteral + pelvicalyceal dilatation in solitary functioning kidney	(Predefined criteria NR)<br>• Obstructive urinary drainage pattern<br>• Equivocal urinary drainage pattern + persisting PM type III dilatation<br>• Recurrent UTI + PM type III dilatation (with improved drainage pattern) in solitary kidney
**Calisti (2008)**	(Predefined criteria NR)<br>• Sepsis<br>• Renal failure in solitary kidney	• Recurrent UTI under CAP<br>• DRF↓ ≥5% during follow-up
**Di Renzo (2013)**	(Predefined criteria NR)<br>• Urosepsis<br>• Other symptoms, not specified<br>• Severe urinary tract dilatation + DRF↓ + obstructive urinary drainage pattern<br>• Severe bilateral pelvicalyceal dilatation<br>• Severe pelvicalyceal dilatation in solitary kidney	• Pelvicalyceal dilatation SFU IV + obstructive urinary drainage pattern or persistent symptoms or DRF↓ ≥5% during follow-up
**Gimpel (2010)**	(Predefined criteria NR)<br>• Initial obstructive (or equivocal) urinary drainage pattern	• Proven relevant obstruction (renogram)<br>• Major complications
**Liu (1994)**	(Predefined criteria NR)<br>• Markedly impaired initial DRF	• Symptomatic UTI under CAP<br>• DRF↓ to <40% or >10% during follow-up
**Oliveira (2000)**	NR	NR
**Stehr (2002)**	• Initial obstructive urinary drainage + DRF <40%	• Normal DRF + at least equivocal obstructive urinary drainage with no improvement or deterioration of urinary drainage and/or DRF↓ during follow-up<br>• Any symptoms (e. g. UTI, calculus)

If generally valid, predefined criteria for surgical intervention were not reported, individual reasons as reported for patients treated operatively were extracted.

Abbreviations: CAP, continuous antibiotic prophylaxis; DRF, differential renal function; NR, not reported; PM, primary megaureter; SFU, Society for Fetal Urology; UTI, urinary tract infection.

Appendix 8: Outcome assessment in the included studies

**Included studies**			**Outcome parameters** (reported for renal units)<br>**R = Resolution / P = Persistence / I = Improvement / D = Deterioration**				
**Study**	**Study cohort**		**Sonographic findings**		**Functional imaging**<br>(^{99m}Tc-DMSA / ^{99m}Tc-DTPA / ^{99m}Tc-MAG3 scintigraphy)		**Secondary surgery**
	**Eligible renal units (review), n**	**Follow-up, months**	**Ureteral dilatation, n**	**Pelvicalyceal dilatation, n**	**Differential renal function (DRF), n**	**Urinary drainage, n**	**n (%)**
**Antón-Pacheco Sanchez (1995)**<br>- Initially 23 patients; 26 renal units	26<br>(23 patients)	Range 12-96	General information without precise information on ureteral and pelvicalyceal dilatation<br>R: 7<br>P: Mild/moderate hydrouereteronephrosis: 17<br>Ureteral dilatation: 2<br>I: NR<br>D: NR		≥40%: 26<br>D: 0	R: 8 (type I)<br>P / I: not separable; overall 17 (type IIIa)<br>D: 1 (type II)<br>→ types according to O`Reilly grading	1 (4%)
**Arena (1998)**<br>- Initially 22 patients; 24 renal units<br>- 1 renal unit excluded (primary surgery)	23<br>(21 patients)	Range 6-54	General information without precise information on ureteral and pelvicalyceal dilatation<br>R: 12<br>P: 4<br>I: 7<br>D: 0		>40%: 22<br>D: 0	Data not separable	4 (17%)
**Calisti (2008)**<br>- Initially 37 patients; 50 renal units	50<br>(37 patients)	Mean 26<br>Range 12-96	General information without precise information on ureteral and pelvicalyceal dilatation<br>R: 46<br>P: NR<br>D: NR		General information: No deterioration in resolved cases<br>D with DRF↓ ≥5%: in at least one of 4 cases; data not precisely reported, but stated as indication for secondary surgery<br>(Mean DRF in 4 renal units with secondary surgery: 34%)	NR	4 (8%)

**Included studies**			**Outcome parameters** (reported for renal units)<br>**R = Resolution / P = Persistence / I = Improvement / D = Deterioration**				
**Study**	**Study cohort**		**Sonographic findings**		**Functional imaging**<br>(^{99m}Tc-DMSA / ^{99m}Tc-DTPA / ^{99m}Tc-MAG3 scintigraphy)		**Secondary surgery**
	**Eligible renal units (review), n**	**Follow-up, months**	**Ureteral dilatation, n**	**Pelvicalyceal dilatation, n**	**Differential renal function (DRF), n**	**Urinary drainage, n**	**n (%)**
**Di Renzo (2013)**<br>- Initially 75 patients; 88 renal units<br>- 14 renal units excluded (primary surgery)	74<br>(63 patients)	Median 120, Range 60-180	General information without precise information on ureteral and pelvicalyceal dilatation<br>R: 47<br>P / I: not separable, mild hydroureteronephrosis: 7<br>D: NR		>40%: 54 (non-surgically managed)<br>+ at least 9 (secondary surgery; preoperatively assessed)<br>D: 4	Not precisely reported	20 (27%)
**Gimpel (2010)**<br>- Initially 49 patients; 56 renal units<br>- 4 renal units excluded (primary surgery)	52<br>(45 patients)	Mean 47±30<br>Range 12-78	General information without precise information on ureteral and pelvicalyceal dilatation<br>R: 36<br>P / I: not precisely reported<br>D: not precisely reported		Data not precisely separable	(15 renal units not examined)<br>R: 2<br>P: 20<br>I: 7<br>D: 6	9 (17%)
**Liu (1994)**<br>- Initially 53 patients; 67 renal units<br>- 1 renal excluded (primary surgery)	66<br>(52 patients)	Mean 37<br>Range 14-60	(Data only reported for 56 non-surgically managed renal units)<br>R: 23<br>P: 22<br>I: 6<br>D: 5	NR	DRF stable in 23 resolved renal units<br>D: 7	Data not separable	10 (15%)
**Oliveira (2000)**<br>- Initially 8 patients; 11 renal units	11<br>(8 patients)	Median 75.5<br>Mean 53<br>Range 37-124	(n=9; 2 renal units not examined initially)<br>R: 7<br>P / I: 0<br>D: 2	NR	>40%: 11<br>D: 0	R / P: not precisely reported<br>I: 11<br>D: 0	0 (0%)
**Stehr (2002)**<br>- Initially 42 patients; 53 renal units<br>- 1 renal unit excluded (primary surgery)	52<br>(41 patients)	Mean 22.1<br>Range 5-48	NR	Data not separable	DRF stable in all renal units<br>D: 0	R: 5<br>Remaining data not separable	4 (8%)

Abbreviations: DRF, differential renal function; NR, not reported; ^{99m}Tc-DMSA, Technetium-99m-dimercaptosuccinyl-acid; ^{99m}Tc-DTPA, Technetium-99m-diethylenetriamine-pentaacetic-acid; ^{99m}Tc-MAG3, Technetium-99m-mercaptoacetyltriglycine

Appendix 9: Differential renal function in patients undergoing surgical intervention over time

Included studies	Primary surgery<br>(Data given for renal units)		Secondary surgery<br>(Data given for renal units)	
	Preoperative DRF	Postoperative DRF	Preoperative DRF	Postoperative DRF
**Antón-Pacheco Sanchez (1995)**	NA	NA	(n=1)<br>≥40%	(n=1)<br>NR
**Arena (1998)**	(n=1)<br>Not examined (patient died)	(n=1)<br>NA	(n=4)<br>1: 75%*<br>2: 44%<br>3: 100%*<br>4: 42%	(n=4)<br>1: NR<br>2: 42%<br>3: NR<br>4: 42%
**Calisti (2008)**	NA	NA	(n=4)<br>Mean DRF at diagnosis: 36%<br>Mean DRF at surgery: 34%	(n=4)<br>No improvement
**Di Renzo (2013)**	(n=14)<br>Data not separable; some cases with DRF↓, not precisely reported	(n=14)<br>NR	(n=20)<br>1: <40%<br>2: 50%<br>3: 46%<br>4: 50%<br>5: 50%<br>6: 40%<br>7: 50%<br>8: <40%<br>9: NR<br>10: 50%<br>11: 50%<br>12: 60%<br>13-20: NR	(n=20)<br>1: 37%<br>2: 50%<br>3: 45%<br>4: 50%<br>5: 50%<br>6: 40%<br>7: 47%<br>8: 38%<br>9: 46%<br>10: 50%<br>11: 50%<br>12: 60%<br>13-20: NR
**Gimpel (2010)**	(n=4)<br>Data not separable	(n=4)<br>Data not separable	(n=9)<br>Data not separable	(n=9)<br>Data not separable
**Liu (1994)**	(n=1)<br>10%	(n=1)<br>NR	(n=10)<br>1: "stable"**<br>2: "stable"**<br>3: "stable"**<br>4: solitary kidney, radionuclide uptake ↓<br>Initial mean DRF in further 6: 45.3% (range, 39-56%)<br>Preoperative mean DRF in those: 32.8% (range, 26-39%)	(n=10)<br>1: "unchanged"**<br>2: "unchanged"**<br>3: "unchanged"**<br>4: NR<br>Data not Separable<br>8 renal units (including primary and secondary surgery):<br>4 renal units: "stable"<br>4 renal units: "regain"

**Oliveira (2000)**	NA	NA	NA	NA
**Stehr (2002)**	(n=1)<br><br>DRF↓, not precisely reported	(n=1)<br><br>NR	(n=4)<br><br>1: 38%<br>2: 39%<br>3: NR***<br>4: 39%	(n=4)<br><br>1: NR<br>2: NR<br>3: NR<br>4: NR

The timing of postoperative DRF assessment was not specified by the included studies.

*contralateral dysplasia
**operated due to urinary tract infection(s)
***operated due to urolithiasis

Abbreviations: DRF, differential renal function; NA, not applicable (no primary or secondary surgical interventions); NR, not reported

Appendix 10: Symptoms in non-refluxing primary megaureter

**Included studies**	**Symptoms and complications** (provided for number of patients)							
	**Urinary tract infection**	**Urolithiasis**	**Arterial hypertension**	**Flank / abdominal pain**	**Acute renal failure**	**Chronically impaired glomerular kidney function**	**Proteinuria**	**Other**
**Antón-Pacheco Sanchez (1995)**	Reported, data not separable	NR	NR	Reported, data not separable	NR	0/23	NR	Vomiting Hematuria<br>Data not separable
**Arena (1998)**	3/22	NR	NR	NR	1/22<br>(Death during neonatal period)	0/21	NR	NR
**Calisti (2008)**	Reported, data not separable	NR	NR	NR	NR<br>(Initial cohort: 2/54, single kidney)	0/37	NR	NR
**Di Renzo (2013)**	Reported, data not separable	NR	NR	Reported, data not separable	NR	NR	NR	NR
**Gimpel (2010)**	33/49<br>→ 17/49 >1 event<br>(2/33: contralateral VUR)	NR	1/49<br>(Nephrectomy due to DRF↓ + treatment resistant arterial hypertension)	1/49	NR	0/49	NR	NR
**Liu (1994)**	3/53	NR	NR	NR	NR	NR	NR	NR
**Oliveira (2000)**	NR	NR	2/8	NR	NR	0/8	NR	NR
**Stehr (2002)**	13/42	1/42	NR	2/42	NR	0/42	NR	NR

Abbreviations: DRF, differential renal function; NR, not reported; VUR, vesicoureteral reflux

Appendix 11: Sensitivity analyses regarding the pooled prevalence of resolution in non-refluxing primary megaureter

Figure 11.1: Studies excluding renal units with suspected obstruction or with missing information on urinary drainage

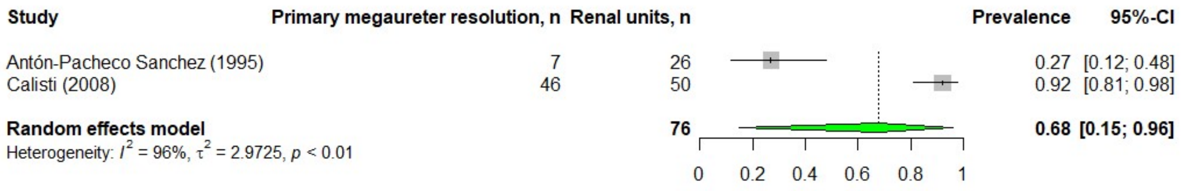

Figure 11.2: Studies including all types of non-refluxing primary megaureter in terms of urinary drainage (studies without precise reporting of this data excluded)

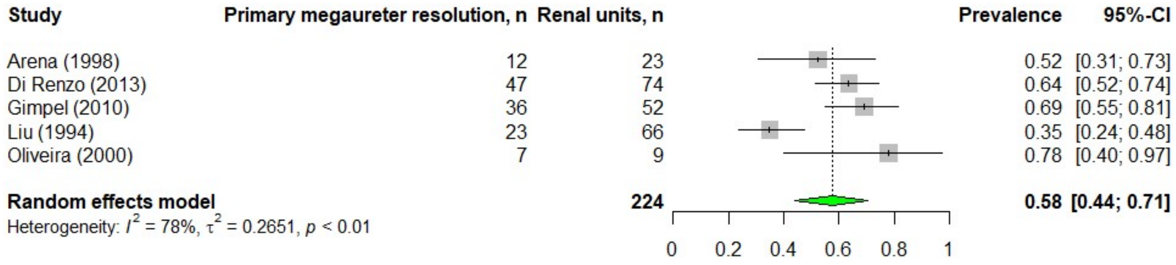

Figure 11.3: Studies with <30 renal units

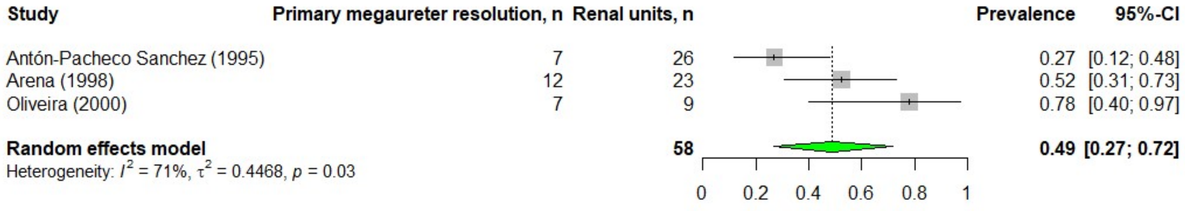

Figure 11.4: Studies with ≥50 renal units

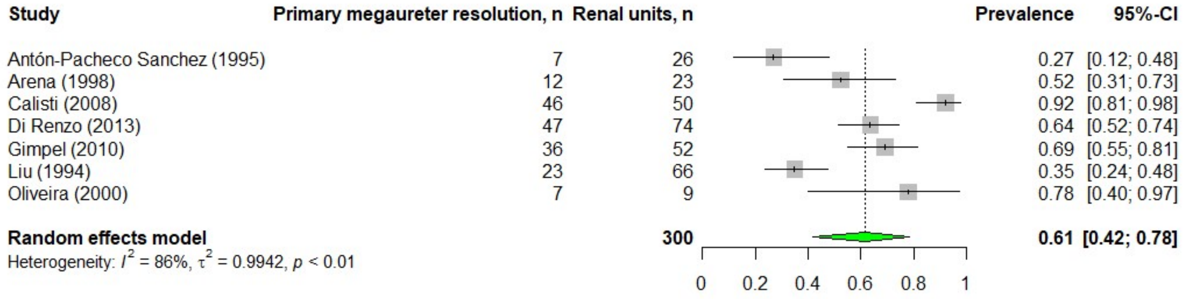

Figure 11.5: Studies published beyond 2000

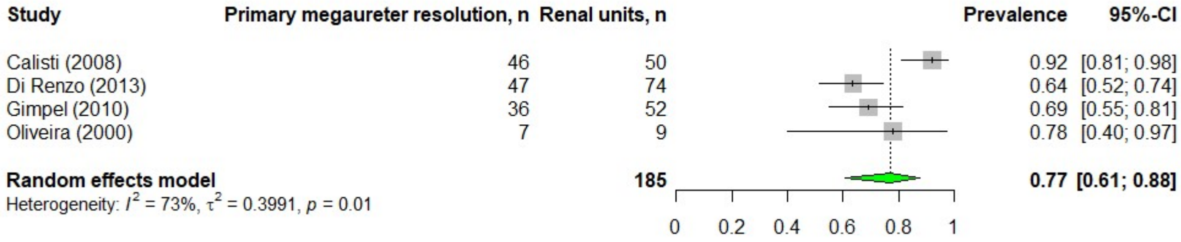

Figure 11.6: Studies with follow-up ≥12 months

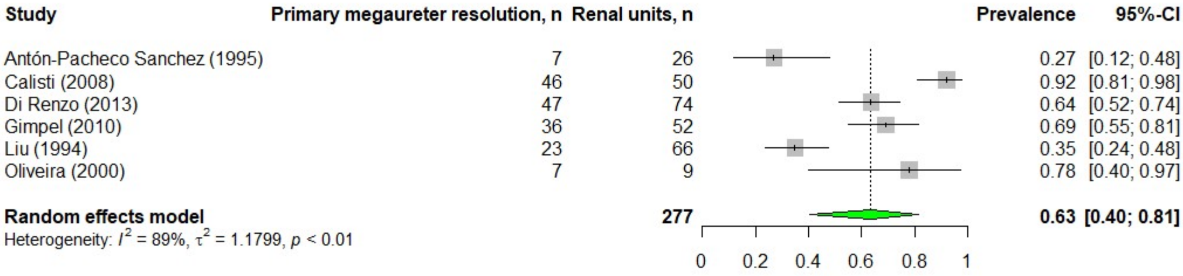

Appendix 12: Sensitivity analyses regarding the pooled prevalence of secondary surgery in non-refluxing primary megaureter

Figure 12.1: Studies excluding renal units with suspected obstruction or with missing information on urinary drainage

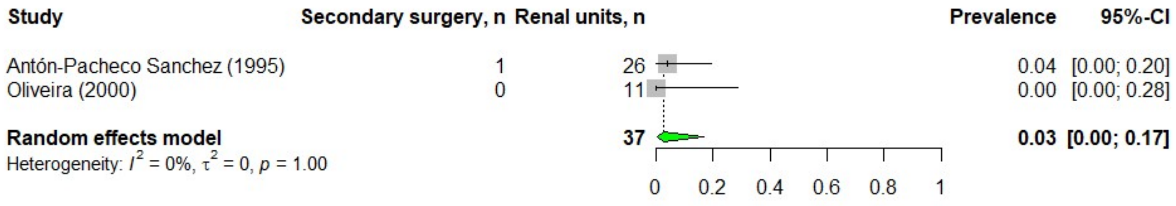

Figure 12.2: Studies including all types of non-refluxing primary megaureter in terms of urinary drainage (studies without precise reporting of this data excluded)

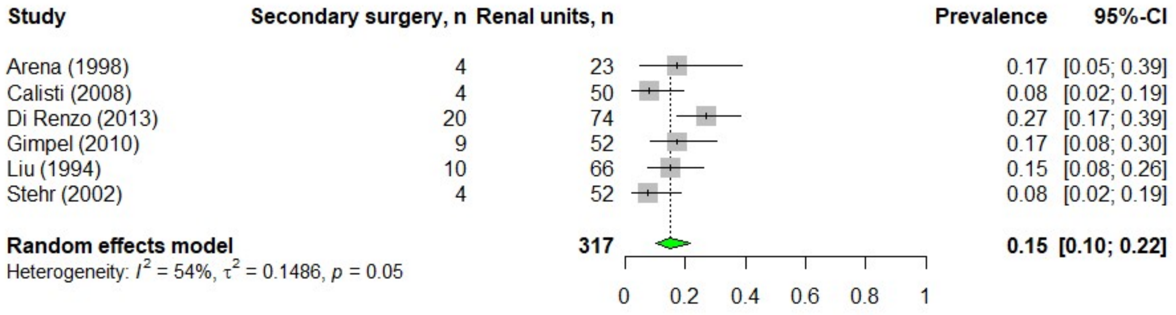

Figure 12.3: Studies with <30 renal units

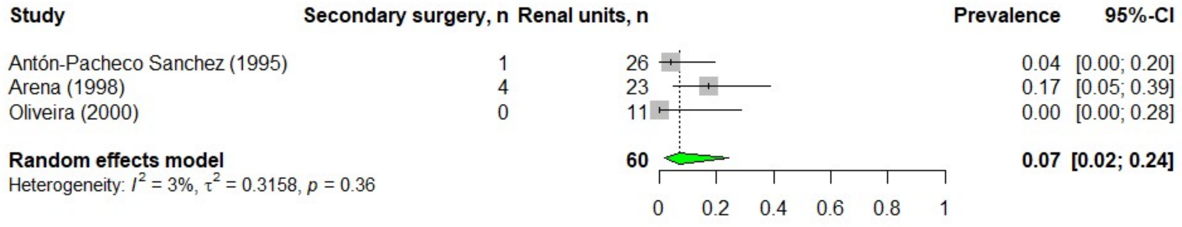

Figure 12.4: Studies with ≥50 renal units

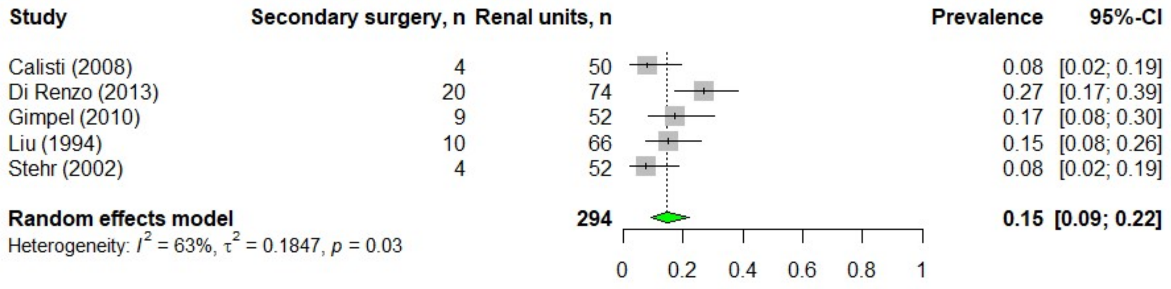

Figure 12.5: Studies published beyond 2000

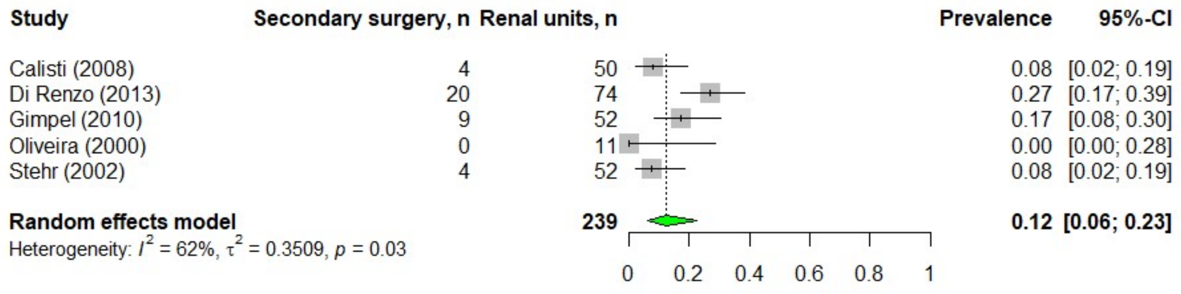

Figure 12.6: Studies including follow-up <12 months

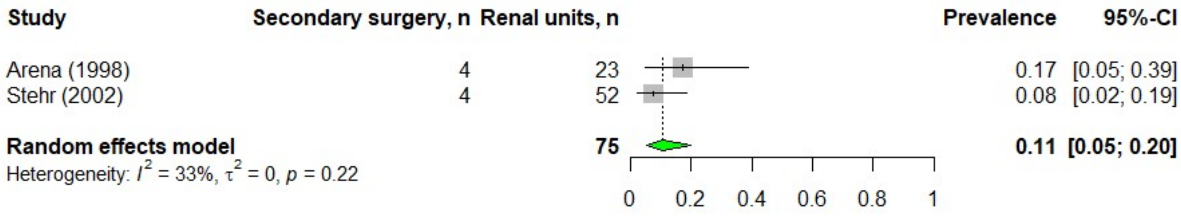

Figure 12.7: Studies with follow-up ≥12 months

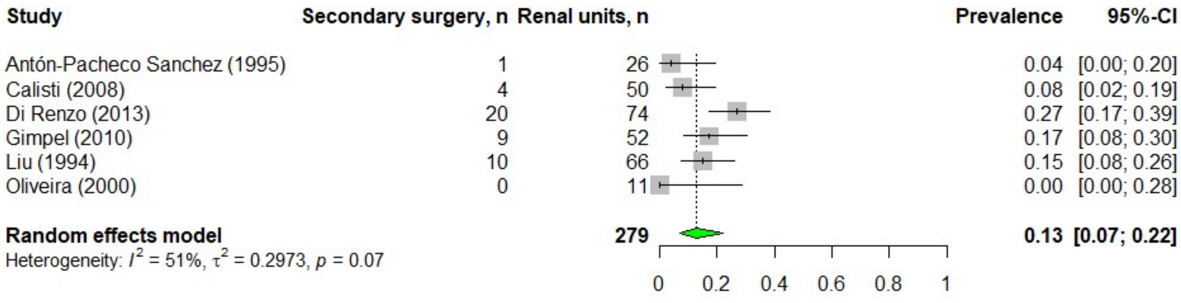

Appendix 13: Funnel plot regarding the outcome resolution

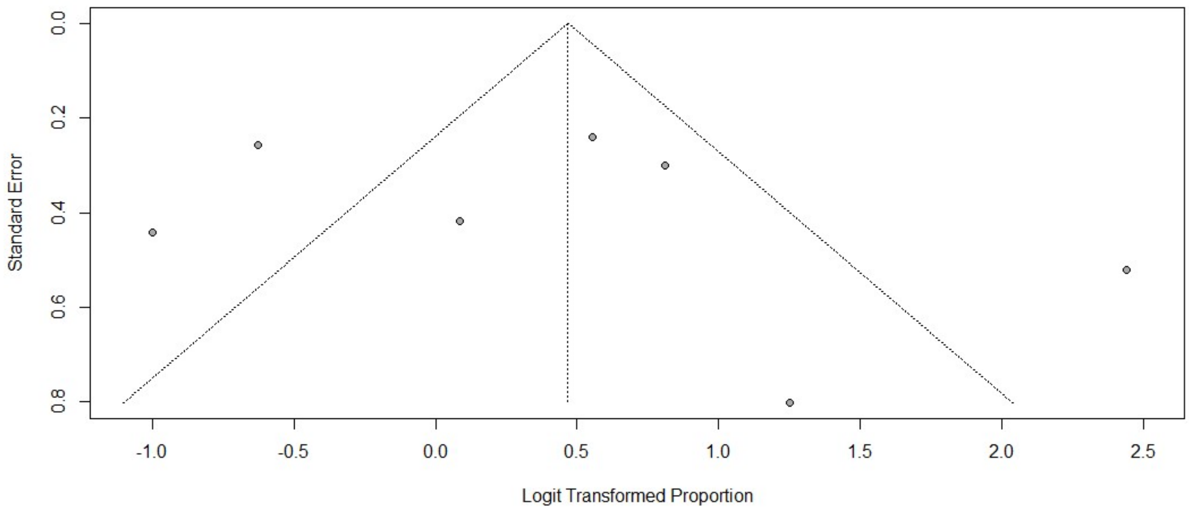

Appendix 14: Funnel plot regarding the outcome secondary surgery

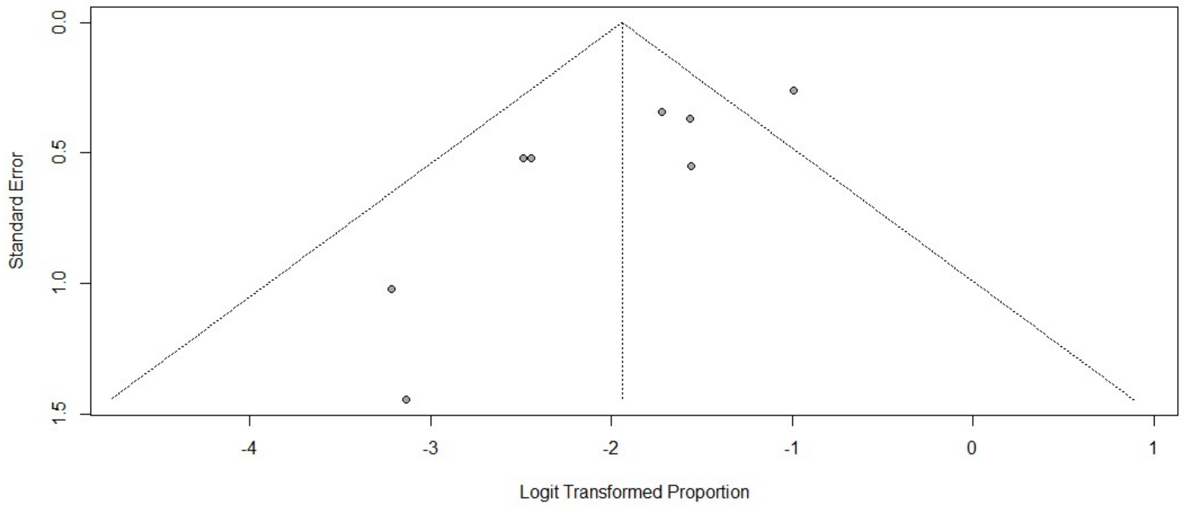

References

1. Anton-Pacheco Sanchez J, Gomez Fraile A, Aransay Brantot A, Lopez Vazquez F, Encinas Goenechea A (1995) Diuresis renography in the diagnosis and follow-up of nonobstructive primary megaureter. Eur J Pediatr Surg 5:338-341. https://doi.org/10.1055/s-2008-1066238
2. Pfister RC, Hendren WH (1978) Primary megaureter in children and adults. Clinical and pathophysiologic features of 150 ureters. Urology 12:160-176. https://doi.org/10.1016/0090-4295(78)90327-8
3. O'Reilly PH, Lawson RS, Shields RA, Testa HJ (1979) Idiopathic hydronephrosis--the diuresis renogram: a new non-invasive method of assessing equivocal pelvioureteral junction obstruction. J Urol 121:153-155. https://doi.org/10.1016/s0022-5347(17)56703-8
4. O'Reilly PH, Testa HJ, Lawson RS, Farrar DJ, Edwards EC (1978) Diuresis renography in equivocal urinary tract obstruction. Br J Urol 50:76-80. https://doi.org/10.1111/j.1464-410x.1978.tb03030.x
5. Arena F, Baldari S, Proietto F, Centorrino A, Scalfari G, Romeo G (1998) Conservative treatment in primary neonatal megaureter. Eur J Pediatr Surg 8:347-351. https://doi.org/10.1055/s-2008-1071230
6. Beurton D (1983) [Primary obstructive megaureter in children and adults]. J Urol (Paris) 89:375-457.
7. Calisti A, Oriolo L, Perrotta ML, Spagnol L, Fabbri R (2008) The fate of prenatally diagnosed primary nonrefluxing megaureter: do we have reliable predictors for spontaneous resolution? Urology 72:309-312. https://doi.org/10.1016/j.urology.2008.02.032
8. Fernbach SK, Maizels M, Conway JJ (1993) Ultrasound grading of hydronephrosis: introduction to the system used by the Society for Fetal Urology. Pediatr Radiol 23:478-480. https://doi.org/10.1007/BF02012459
9. Di Renzo D, Aguiar L, Cascini V, Di Nicola M, McCarten KM, Ellsworth PI, Chiesa PL, Caldamone AA (2013) Long-term followup of primary nonrefluxing megaureter. J Urol 190:1021-1026. https://doi.org/10.1016/j.juro.2013.03.008
10. Gimpel C, Masioniene L, Djakovic N, Schenk JP, Haberkorn U, Tonshoff B, Schaefer F (2010) Complications and long-term outcome of primary obstructive megaureter in childhood. Pediatr Nephrol 25:1679-1686. https://doi.org/10.1007/s00467-010-1523-0
11. Hofmann V, Deeg KH, Hoyer PF (1996) Ultraschalldiagnostik in Pädiatrie und Kinderchirurgie. Thieme, Stuttgart, New York, p 382.
12. Liu HY, Dhillon HK, Yeung CK, Diamond DA, Duffy PG, Ransley PG (1994) Clinical outcome and management of prenatally diagnosed primary megaureters. J Urol 152:614-617. https://doi.org/10.1016/s0022-5347(17)32664-2
13. Oliveira EA, Diniz JS, Rabelo EA, Silva JM, Pereira AK, Filgueiras MT, Soares FM, Sansoni RF (2000) Primary megaureter detected by prenatal ultrasonography: conservative management and prolonged follow-up. Int Urol Nephrol 32:13-18. https://doi.org/10.1023/a:1007101227302
14. Stehr M, Metzger R, Schuster T, Porn U, Dietz HG (2002) Management of the primary obstructed megaureter (POM) and indication for operative treatment. Eur J Pediatr Surg 12:32-37. https://doi.org/10.1055/s-2002-25088
